# Genomic data analysis workflows for tumors from patient-derived xenografts (PDXs): challenges and guidelines

**DOI:** 10.1186/s12920-019-0551-2

**Published:** 2019-07-01

**Authors:** Xing Yi Woo, Anuj Srivastava, Joel H. Graber, Vinod Yadav, Vishal Kumar Sarsani, Al Simons, Glen Beane, Stephen Grubb, Guruprasad Ananda, Rangjiao Liu, Grace Stafford, Jeffrey H. Chuang, Susan D. Airhart, R. Krishna Murthy Karuturi, Joshy George, Carol J. Bult

**Affiliations:** 1 The Jackson Laboratory for Genomic Medicine, Farmington, CT 06030 USA; 2 The Jackson Laboratory for Mammalian Genetics, Bar Harbor, ME 04609 USA; 3 MDI Biological Laboratory, Bar Harbor, ME 04609 USA; 4 Present Address: University of Massachusetts, Amherst, MA 01003 USA; 5 Present Address: Massachusetts Institute of Technology, Cambridge, MA 02139 USA; 6 Present Address: Novogene Corporation, Rockville, MD 20850 USA

**Keywords:** Patient-derived xenografts, DNA sequencing, RNA sequencing, SNP array, Somatic mutation, Gene expression, Copy number alterations, Mouse stroma, Bioinformatic analysis

## Abstract

**Background:**

Patient-derived xenograft (PDX) models are in vivo models of human cancer that have been used for translational cancer research and therapy selection for individual patients. The Jackson Laboratory (JAX) PDX resource comprises 455 models originating from 34 different primary sites (as of 05/08/2019). The models undergo rigorous quality control and are genomically characterized to identify somatic mutations, copy number alterations, and transcriptional profiles. Bioinformatics workflows for analyzing genomic data obtained from human tumors engrafted in a mouse host (i.e., Patient-Derived Xenografts; PDXs) must address challenges such as discriminating between mouse and human sequence reads and accurately identifying somatic mutations and copy number alterations when paired non-tumor DNA from the patient is not available for comparison.

**Results:**

We report here data analysis workflows and guidelines that address these challenges and achieve reliable identification of somatic mutations, copy number alterations, and transcriptomic profiles of tumors from PDX models that lack genomic data from paired non-tumor tissue for comparison. Our workflows incorporate commonly used software and public databases but are tailored to address the specific challenges of PDX genomics data analysis through parameter tuning and customized data filters and result in improved accuracy for the detection of somatic alterations in PDX models. We also report a gene expression-based classifier that can identify EBV-transformed tumors. We validated our analytical approaches using data simulations and demonstrated the overall concordance of the genomic properties of xenograft tumors with data from primary human tumors in The Cancer Genome Atlas (TCGA).

**Conclusions:**

The analysis workflows that we have developed to accurately predict somatic profiles of tumors from PDX models that lack normal tissue for comparison enable the identification of the key oncogenic genomic and expression signatures to support model selection and/or biomarker development in therapeutic studies. A reference implementation of our analysis recommendations is available at https://github.com/TheJacksonLaboratory/PDX-Analysis-Workflows.

**Electronic supplementary material:**

The online version of this article (10.1186/s12920-019-0551-2) contains supplementary material, which is available to authorized users.

## Background

Patient-derived xenograft (PDX) models are in vivo models of human cancer that have been used for translational cancer research and therapy selection for individual patients [1–8]. Previous studies have demonstrated human tumors engrafted in mouse hosts retain therapeutically relevant genomic aberrations found in the original patient tumor [3, 9, 10] and that treatment responses of tumor-bearing mice typically reflect the responses observed in patients [6, 11]. PDXs have been used successfully as a platform for pre-clinical drug screens [6, 7, 11], to facilitate the development of potential biomarkers of drug response and resistance [6, 7, 12], and to select appropriate therapeutic regimens for individual patients [9].

**Fig. 1:**
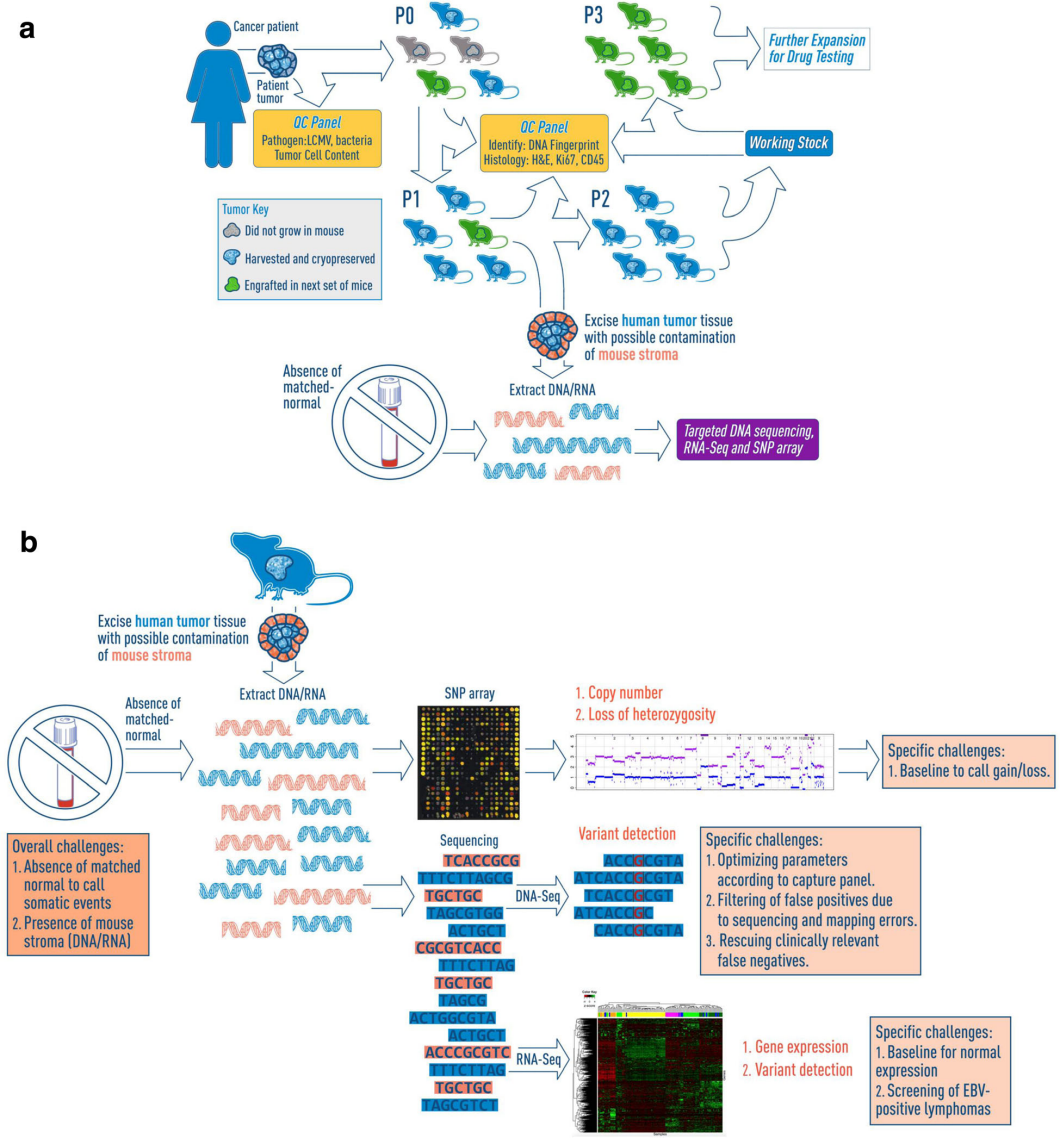
Overview of Patient-Derived Xenograft (PDX) model generation and genomic characterization at The Jackson Laboratory (JAX). **a** Schematic overview of PDX model generation and characterization for the JAX PDX resource. JAX has generated, clinically annotated, and genomically characterized 455 PDX cancer models originating from 34 different primary sites (as of 05/08/2019) using the immunodeficient NOD.Cg-*Prkdc*^*scid*^ *Il2rg*^*tm1Wjl*^/SzJ (aka, NSG™) mouse as the host strain. **b** Schematic of the genomic characterization of PDX models (see Methods for details). The three primary genomic characterization methods are: 1) somatic mutations using the JAX Cancer Treatment Profile™ (CTP, https://www.jax.org/clinical-genomics/clinical-offerings/jax-cancer-treatment-profile), the Illumina TruSeq™ panel or whole-exome sequencing, 2) DNA copy-number variation using Affymetrix SNP 6.0 arrays, and 3) gene expression profiles from Affymetrix microarrays or RNA sequencing (Illumina HiSeq)

The Jackson Laboratory (JAX) PDX resource comprises 455 PDX cancer models originating from 34 different primary sites (as of 05/08/2019, Table S14). The models undergo rigorous quality control and are genomically characterized to identify somatic mutations, copy number alterations, and transcriptional profiles (Fig. 1). To date, over 100 models in the resource have been assessed for their response to cytotoxic and/or targeted therapeutic agents. The integration of results from dosing studies with genomic data for the models has been successfully applied to the identification of novel genomic biomarkers associated with treatment responses [13].

Four major challenges need to be addressed to genomically characterize human tumors engrafted in a mouse host. First, mouse sequences must be removed from a data set prior to data analysis. Nucleic acids extracted from engrafted tumors include both mouse and human sequences because human stroma is replaced by mouse cells during tumor engraftment [14]. As the protein-coding regions of the mouse and human genomes are 85% identical on average [15]; there is a high risk of introducing false positive (FP) variants resulting from mouse sequences aligning to a reference human genome [16–18]. Second, a baseline normal must be created to identify aberrations that are likely somatic. Paired normal samples are not available for the majority of JAX PDXs because the tumor tissue used to create the models was material that remained following clinical pathology assessment (i.e., tumors were not collected specifically for xenograft model creation). The absence of genomic data from paired normal tissue complicates the process of distinguishing germline variants from somatic alterations (point mutations, indels, and copy number alterations) in the tumor [19–22]. Third, systematic errors in sequencing and alignment can lead to FP variant calls and require customized filtering logic in computational workflows [23–25]. Finally, care must be taken to ensure the engrafted tumors in PDXs match the expected cancer type. Some strains of immunodeficient host mice are susceptible to forming B-cell human lymphomas during engraftment due to Epstein-Barr virus (EBV)-associated lymphomagenesis [26–30]. Identifying PDX tumors that arise from EBV transformation is critical to the model integrity and to meaningful genomic data analysis.

Here we describe bioinformatics analysis workflows and guidelines (https://github.com/TheJacksonLaboratory/PDX-Analysis-Workflows) that we developed specifically for the analysis of genomic data generated from PDX tumors. Our workflows incorporate commonly used software and public databases but are tailored to address the specific challenges of PDX genomics data analysis through parameter tuning and customized data filters and result in improved accuracy for the detection of somatic alterations in PDX models relative to analyses that lacked custom filters. We also report a gene expression-based classifier that can identify EBV-transformed tumors. Finally, to demonstrate the effectiveness of our workflows, we show the overall concordance of the genomic and transcriptomic profiles of the PDX models in the JAX PDX resource with relevant tumor types from The Cancer Genome Atlas (TCGA).

## Methods

### Genomic and transcriptomic profiling of engrafted tumors

#### DNA sequencing

Flash frozen tissues were pulverized using a Bessman Tissue Pulverizer (Spectrum Chemical) and homogenized in Nuclei Lysis Buffer (Promega) using a gentleMACS dissociator (Miltenyi Biotec Inc). DNA was isolated using the Wizard Genomic DNA Purification Kit (Promega) according to manufacturer’s protocols. DNA quality and concentration were assessed using a Nanodrop 2000 spectrophotometer (Thermo Scientific), a Qubit dsDNA BR Assay Kit on a Qubit Fluorometer (Thermo Scientific), and the Genomic DNA ScreenTape on a 4200 TapeStation (Agilent Technologies). Libraries were prepared using the Hyper Prep Kit (KAPA Biosystems) and SureSelectXT Target Enrichment System with the JAX Cancer Treatment Profile (CTP) targeted panel of 358 related genes (Agilent Technologies) [31, 32], according to the manufacturer’s instructions. Briefly, the protocol entails shearing the DNA using the Covaris E220 Focused-ultrasonicator (Covaris), ligating Illumina specific adapters, and PCR amplification. Amplified DNA libraries are then hybridized to the CTP probes, amplified using indexed primers, and checked for quality and concentration using the High Sensitivity D5000 ScreenTape (Agilent Technologies) and Qubit dsDNA HS Assay Kit (Thermo Scientific). Libraries were pooled and sequenced 150 bp paired-end on the NextSeq 500 (Illumina) using NextSeq v2 reagents (Illumina).

#### RNA sequencing

Tissues preserved in RNAlater were homogenized in TRIzol (ThermoFisher Scientific) using a gentleMACS dissociator (Miltenyi Biotec Inc). Total RNA was isolated using the miRNeasy Mini kit (Qiagen) according to manufacturer’s protocols, including the optional DNase digest step. RNA quality and concentration were assessed using the RNA 6000 Nano LabChip assay on the 2100 Bioanalyzer instrument and Nanodrop 2000 spectrophotometer (Thermo Scientific). Prior to 2016, non-stranded libraries were constructed using TruSeq RNA Library Prep Kit v2 (Illumina). Stranded libraries were prepared using the KAPA mRNA HyperPrep Kit (KAPA Biosystems), according to the manufacturer’s instructions. Briefly, the protocol entails isolation of polyA containing mRNA using oligo-dT magnetic beads, RNA fragmentation, first and second strand cDNA synthesis, ligation of Illumina-specific adapters containing a unique barcode sequence for each library, and PCR amplification. Libraries were checked for quality and concentration using the DNA 1000 assay (Agilent Technologies) and quantitative PCR (KAPA Biosystems), according to the manufacturers’ instructions. Libraries were pooled and sequenced 75 bp paired-end on the NextSeq 500 (Illumina) using NextSeq High Output Kit v2 reagents (Illumina), or 100 bp paired-end on the HiSeq2500 (Illumina) using TruSeq SBS v3 reagents (Illumina).

#### SNP array

DNA samples were sent to the Genotyping Core at the Hussman Institute for Human Genomics (University of Miami) for genotyping on the Genome-Wide Human SNP Array 6.0 (Affymetrix). Quality control on the CEL files was carried out using the standard Contrast QC metric from the Affymetrix Genome Wide SNP 6.0 array manual.

### Somatic point mutation and indel calling workflow

#### Preprocessing and removal of mouse reads

DNA sequence data generated from PDX tumors underwent initial data processing as follows: (i) sequence reads with 70% of the bases having a quality score < 30 (Q30) were discarded, (ii) bases with quality scores less than Q30 were trimmed from the 3′ end of the read, (iii) sequence reads with < 70% of bases remain after trimming were discarded, (iv) both reads from pair-end sequencing were discarded if either read was discarded. If < 50% of the total reads remained following the preprocessing steps, the sample was removed from the analysis. Following the initial data processing step described above, mouse reads were identified and filtered out using Xenome v1.0.0 [16]. Only read pairs with both reads classified as human were included in further analyses.

Sequence reads that passed all pre-processing steps were mapped to the reference human genome (build GRCh38.p5 with 262 alternate loci) using the BWA-MEM alignment tool with ALT-Aware mapping (Additional file 1: Text S5) [33–35]. Because low sequence coverage leads to poor sensitivity in variant calling, samples with less than 75% of the target region covered at least at ≥100X by human reads were excluded from further analysis.

#### Variant calling

The GATK best practices workflow (https://software.broadinstitute.org/gatk/best-practices/) using the UnifiedGenotyper, was used for variant discovery analysis [36–38], which is comprised of the following steps: (i) sorting the SAM/BAM file by coordinate, (ii) removing duplicates to mitigate biases introduced by library preparation steps such as PCR amplification by Picard (https://broadinstitute.github.io/picard/), and (iii) recalibrating the base quality scores as the variant calling algorithms rely heavily on the quality scores assigned to the individual base calls in each sequence read. Pindel [39] was also incorporated into the workflow to call indels that have been missed by the GATK UnifiedGenotyper.

#### Quality filtering of variants for targeted sequencing

High quality variants from both variant callers in the PDX samples were obtained based on GATK hard filtering (see below), and have a read depth (DP) of ≥140 and allele frequency (ALT_AF) of ≥5%. These DP and ALT_AF thresholds were optimized using a set of known and validated mutations and samples reported earlier for the JAX CTP targeted panel sequencing at high coverage (average 941 X) [31, 32]. The parameters for GATK hard filtering [40] were set as default as recommended by GATK best practices (https://software.broadinstitute.org/gatk/documentation/tooldocs/current/, https://software.broadinstitute.org/gatk/best-practices/):

(i) for point mutations, QD < 2.0, FS > 60.0, MQ < 40.0, MQRankSum < − 12.5, ReadPosRankSum < − 8.0.

(ii) for indels, QD < 2.0, FS > 200.0, ReadPosRankSum < − 20.0.

In addition, we verified that these default thresholds were able to detect all the known mutations in the CTP samples [31]. The average number of variants before and after quality filtering across the CTP samples is shown Additional file 1: Table S4.

#### Annotation of variants

Variants were annotated for their effect (gene, consequence, amino acid change, etc.) using SnpEff v4.3 [41] based on gene annotations from Ensembl (version 84) and information from COSMIC version 80 [42], dbSNP build 144 [43]. The observed variant allele frequency in the 1000 Genomes Project [44] and ExAC version 0.3 [31, 45] databases were obtained using SnpSift tool by utilizing dbNSFP3.2a.txt database. We further annotated each variant with 1) known or predicted gain or loss of protein function, 2) potential treatment approach for any cancer type and 3) drug sensitivity and resistance effects in clinical or preclinical studies, based on curated clinical information from the JAX clinical knowledge base (CKB, https://ckbhome.jax.org/) [46, 47] via direct integration of our internal database of PDX data with the JAX CKB database. The JAX CKB contains annotations for 28,362 variants in 1320 genes (as of 05/03/2019). The average number of variants annotated to be clinically relevant across the CTP samples is shown in Additional file 1: Table S4.

#### Filtering of germline variants

Since normal samples from patients whose tumors were used to generate the PDX models were unavailable in most cases, we generated a dataset of putative human germline variants using data from several public resources: (i) dbSNP, (ii) 1000 Genomes Project, (iii) ExAC database with MAF ≥1%, and (iv) a compendium of variants from 20 normal human blood samples available in JAX (Additional file 1: Text S1) that were prepped and sequenced on the CTP panel using the same protocol as the PDX samples, with a frequency of 2/20 in normal samples or 1/20 in normal samples and 2/20 in PDX models. The number of variants in each of these databases are shown in Additional file 1: Table S3. The variants identified via GATK and Pindel in the PDX model tumors were annotated as germline and filtered out of the model’s somatic mutation calls if they were present in our aggregated dataset of putative germline variants and had allele frequencies between 40 to 60% or more than 90%.

#### Filtering putative false positives

Variants not in our aggregated dataset of putative germline variants described above but occurred at a frequency of 25% or greater across all PDX models (*n* = 236) were considered to be putative false positive (FP) mutations. The rationale for this data filtering step was based on our observation that the maximum recurrent frequency of somatic mutated base positions was 6% across a compendium of TCGA tumor samples (*n* = 3576, 9 tumor types that were also represented in the PDX model). Thus, we would expect that any mutated loci recurring across PDX samples at significantly higher rates to likely be FP. Systematic technical errors in sequencing and/or mapping are possible explanations for the common recurrent non-somatic mutations identified PDX models.

#### Rescuing false negative variants

An exception to the germline and false positives exclusion process was made for variants from GATK that were annotated as clinically relevant in JAX CKB. We rescued any filtered variants that were curated into the proprietary JAX-Clinical Knowledgebase (CKB, https://ckbhome.jax.org/) with 1) known or predicted gain or loss of protein function, 2) potential treatment approach for any cancer type and 3) drug sensitivity and resistance effects in clinical or preclinical studies. In addition, as Pindel results contained a large number of FPs, we only included those that were present in the JAX-CKB by the same criteria.

### Benchmarking of PDX somatic mutation workflow

To benchmark the PDX somatic mutation workflow, we generated simulated datasets for five PDX models and nine conditions. The datasets included 1) varying sequencing coverage, 2) spiked-in mutations representative of the different tumor types, and 3) different proportions of spiked-in mouse sequence contamination (Additional file 2: Table S1).

#### Generation of simulated sequence reads

SeqMaker was used to generate simulated sequencing data based on human genome assembly GRCh38.5 with varying sequencing depth, read length, duplication rate, sequencing error and base quality range [48]. Reference sequences were extracted from target region of the CTP panel. Sequence reads for 5 PDX tumor samples were simulated using predicted mutations from PDX models of different cancer types from the CTP dataset to represent different spectrum of mutations, with a range of allele frequency to mimic germline and somatic mutations. For each simulated sample, we generated three technical replicates at 500X, 1000X and 1500X coverage.

Mouse sequencing reads were added in different fractions to the human-specific simulated dataset to mimic mouse contamination observed in PDX models. The mouse reads were extracted from the sequencing data of mouse DNA isolated from fresh spleen tissue of NSG mice on the CTP. For each simulated human-specific sample, we added mouse reads in three proportions (10, 15 and 25% of the total coverage).

#### Calculate sensitivity and specificity of mutation results based on different workflow filters

To evaluate the effect of each filter used in our workflow, we modified the somatic mutation workflow by: (i) omitting Xenome to filter mouse reads, and (ii) mapping to the reference sequence using BWA-MEM. Each modified workflow was used to process each PDX simulated library and each set of results, with and without quality filters, was used to compute the lists of true positive, false positive, true negative and false negative variants. As such, we can calculate the range of sensitivities and specificities of the predicted variants for all the simulated PDX models. We compared the distributions of precision, recall and F1-score (2*(Recall*Precision)/(Recall+Precision)) for different variations of the variant calling workflow on the simulated datasets. Furthermore, we compared the predicted allele frequencies of the true positives of each sample with the input by correlation.

### Gene expression workflow

#### Data processing and expression estimation

Prior to alignment to the human transcriptome, sequences from PDX tumors were processed for sequence quality. Only sequences with base qualities ≥30 over 70% of read length were used in downstream analyses. Quality trimmed reads were then analyzed using the default parameters of Xenome v1.0.0 (k = 25) [16] to separate human, mouse, and ambiguous sequences (i.e., sequences that cannot be reliably classified as mouse or human). Sequence reads that passed the quality and Xenome screening were aligned to a human transcriptome dataset (ENSEMBL version 84) using Bowtie v2.2.0 [49, 50]. Only samples with at least 1 million human reads were retained for expression analysis to ensure reliable quantification of gene expression level for all genes. The 1 million human read cutoff was determined using down-sampling experiments (data not shown). Gene expression estimates were determined using RSEM v1.2.19 [51] (*rsem-calculate-expression*) with default parameters. We further normalized the expression estimate (expected_count from RSEM) using upper quantile normalization of non-zero expected counts and scaling to 1000.

### Classifier for EBV-associated PDX lymphomas

A gene signature for identifying putative EBV-associated lymphomas was derived by calculating the fold change of the average expression of the expressed genes between 20 EBV-associated lymphomas and 100 non-EBV tumors based on the Z-score transformation of upper-quantile normalized RNA-Seq counts (RSEM). 24 up-regulated and 24 downregulated genes, selected based on the highest and lowest fold change values, were used to define the list of classifier genes (Additional file 1: Table S6). We further checked that the expression levels of these classifier genes were consistent among the EBV-associated lymphomas, and were able to cluster the EBV-associated lymphomas separately from other non-lymphoma PDX tumors (Fig. S5). Gene set analysis on the resulting expression vector was performed with GSEA using the GenePattern webserver and default parameters (data not shown). For each PDX sample, the upper-quantile normalized counts from RSEM of the classifier genes were transformed into z-scores using the mean and standard deviation computed across all PDX samples for each gene. Subsequently, a sign corresponding to the direction of regulation in the classifier table was multiplied to each z-score and the sum of these modified z-scores resulted in a single score for each PDX sample. A classifier score of > 3.0 was used to identify a PDX tumor sample as a potential EBV-associated lymphoma.

### Copy number alterations (CNA) workflow

#### Assessing the effects of mouse DNA on SNP array

DNA of the NSG mouse was hybridized on the Affymetrix SNP 6.0 array, and the signal intensity was extracted from the CEL files using Affymetrix Power Tools (apt-cel-extract). The mouse content for each PDX sample was estimated by the mouse reads proportion computed by Xenome of the mutation calling pipeline for the CTP sequencing of the same PDX sample.

#### Single-tumor CNA analysis

PennCNV-Affy and Affymetrix Power Tools [52–54] were used to extract the B-allele frequency (BAF) and Log R Ratio (LRR) from the resulting CEL files of the Affymetrix Human SNP 6.0 array. Due to the absence of paired-normal samples, the allele-specific signal intensity for each PDX tumor were normalized relative to 300 randomly selected sex-matched Affymetrix Human SNP 6.0 array samples obtained from the International HapMap project [55]. The single tumor version of ASCAT 2.4.3 [56], which could infer the necessary germline genotypes from the tumor data, was then used for GC correction, predictions of the heterozygous germline SNPs and estimation of ploidy, tumor content and copy number segments with allele-specific copy number.

#### Tumor-normal CNA analysis

The same normalization steps as the single-tumor analysis were applied in which both tumor and normal CEL files were normalized with 300 HapMap samples, followed by the CNA analysis using the tumor-normal version of ASCAT 2.4.3.

#### Annotation of CNA segments

The resultant copy number segments were annotated with loss of heterozygosity (LOH) and log_2_ ratio of total copy number relative to diploid state (copy number 2) and predicted ploidy from ASCAT. A segment was defined as LOH when the major-allele copy number was ≥0.5 and the minor-allele copy number was ≤0.1. Gene-level copy number and LOH were estimated by intersecting the genome coordinates of copy number segments with genome coordinates of genes (Ensembl version 84; genome assembly GRCh38.5). In cases where a segment boundary was contained within a gene’s coordinates, the most conservative (lowest) estimate of copy number was used to represent the copy number of the entire intact gene, and the gene was annotated with the number of overlapping segments

#### Defining copy number gain and loss

The low-level copy number gain or loss of a gene was defined by the log_2_ ratio of the copy number relative to the average ploidy of the sample or diploid state with a threshold of ±0.4 respectively. We compiled a list of genes with focal copy number alterations that were significantly amplified (*n* = 273) or deleted (*n* = 820) in the 8 tumor types (Additional file 1: Fig. S8 and Table S12) from the GISTIC 2.0 analysis from the TCGA FireBrowse website (http://firebrowse.org/). Using this set of genes, we compared the proportion of genes that would be classified as gain and loss when using different baselines (diploid state 2 or ASCAT predicted ploidy) for PDX models listed in Additional file 1: Table S12.

#### Comparison of copy number alterations with gene expression

Using annotations from the Cancer Gene Census resource [57] we analyzed the relationship between copy number alterations and gene expression using a list of 23 oncogenes that are commonly amplified in cancers and a list of 40 tumor suppressor genes that are commonly deleted in cancers. These genes were classified into copy number states of high-level loss (log2(CN/ploidy) < − 1), normal (− 1 ≤ log_2_(CN/ploidy) ≤ + 1) and high-level gain (log_2_(CN/ploidy) > + 1). The expression fold change of each gene was calculated as the log_2_(TPM + 1) relative to the mean expression across PDX samples with a stringent normal copy number state (− 0.4 ≤ log_2_(CN/ploidy) ≤ 0.4). The significance of expression changes of each gene for the entire PDX resource with copy number gain or loss relative to the normal state was calculated using the Student’s t-Test.

### Comparison between PDX and TCGA data

#### Somatic mutations

We calculated the distribution of mutational load (number of non-silent, coding mutations in exonic regions per sample) of the CTP genes for 6 tumor types with at least 10 models in the PDX resource (colorectal cancer, lung adenocarcinoma, lung squamous cell carcinoma, melanoma, bladder carcinoma and triple-negative breast cancer, Additional file 1: Table S5). MAF files for somatic mutations based on whole-exome sequencing of the TCGA samples of 6 tumor types [58–62] were obtained from TCGA Data Portal and were used to compute the mutation frequency for CTP genes only. The Fisher’s exact test was used to test the significance of overlap of mutated genes between the PDX resource and TCGA patient cohorts for each tumor type. The genes in each PDX resource were considered if they were mutated in at least one sample, while the genes in each TCGA tumor cohort were considered if they were mutated with at least 5% frequency, due to a much larger sample size.

#### Gene expression

6 tumor types with at least 10 models in the PDX resource were selected for comparison with TCGA (colorectal cancer, lung adenocarcinoma, lung squamous cell carcinoma, melanoma, bladder carcinoma and triple-negative breast cancer, Additional file 1: Table S10). The scaled estimate (TPM × 10^− 6^) from the RNA-Seq data of 6 tumor types in TCGA [58–63] were obtained from the TCGA FireBrowse website (http://firebrowse.org/). Non-expressed genes across all tumor types were removed (log_2_(TPM + 1) < 2), and the top 1000 most varying genes based on the variance of their z-scores of log_2_(TPM + 1) across all tumor types were selected to cluster the TCGA samples by hierarchical clustering. These 1000 most varying genes were intersected, by common gene symbols, with the PDX expression data. The symbols of genes that mapped to multiple genomic locations were removed, leaving 993 overlapping genes. These 993 genes were then used to cluster the PDX samples by hierarchical clustering. The frequencies of over-expression and under-expression of each gene is defined by the z-scores of log_2_(TPM + 1) of > + 1 and < − 1 respectively. Correlation of the gene expression frequencies in each tumor type was computed using Pearson correlation. The differential gene expression of each tumor type compared to all other tumor types was computed using limma and edgeR based on expected counts from RSEM with TMM-normalization and voom transformation [64, 65]. Up-regulated (adjusted *p*-value < 0.05, log (fold change) > 1) or down-regulated (adjusted p-value < 0.05, log (fold change) < − 1) genes were obtained for the PDX resource and TCGA patient cohorts separately. The significance of overlap of each set of genes between PDX and TCGA RNA-Seq data was determined using hypergeometric *p*-value.

#### Copy number alterations

Eight tumor types with at least 10 models in the PDX resource (colorectal cancer, lung adenocarcinoma, lung squamous cell carcinoma, melanoma, glioblastoma multiforme, bladder carcinoma, triple-negative breast cancer and ovarian carcinoma, Additional file 1: Table S12) selected to compare with corresponding primary tumors in the TCGA [58–63, 66–68]. For PDX samples, the low-level copy number gain or loss of a gene was defined by the log_2_ ratio of the copy number relative to the average ploidy of the sample (or copy number state 2) with a threshold of ±0.4 respectively. The amplification or deletion calls of each gene for the TCGA samples were provided (loss = − 1, normal = 0, gain = 1) by FireBrowse (http://firebrowse.org/). Using the list of genes with focal copy number alterations that were significantly amplified (*n* = 273) or deleted (*n* = 820) in the 8 tumor types from the GISTIC 2.0 analysis from the TCGA FireBrowse website, we calculated the copy number gain and loss frequencies of these genes for each tumor type in the PDX resource and TCGA cohorts using the respective gain and loss calls.

## Results

### Somatic point mutation and indels

**Fig. 2:**
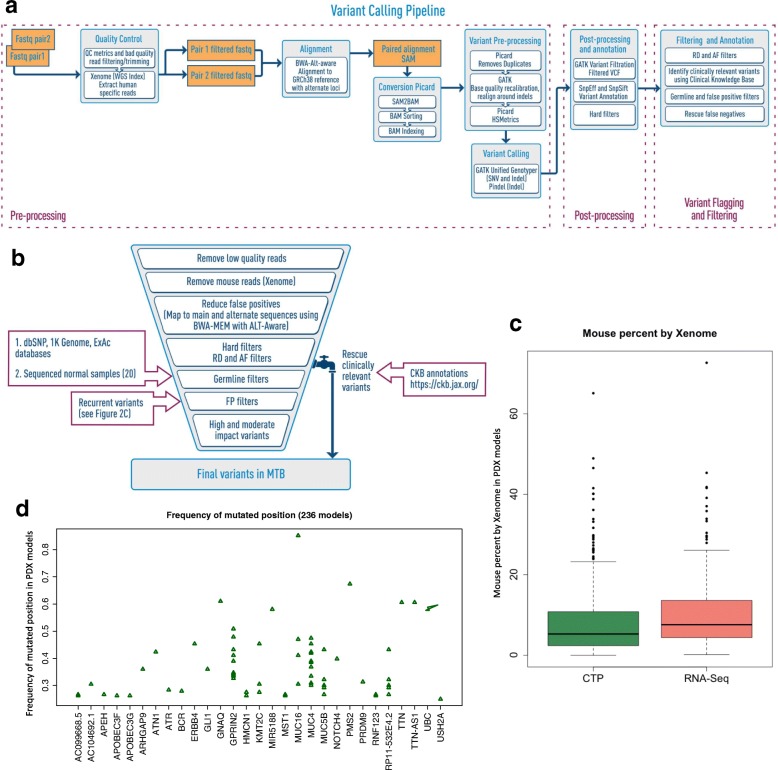
A multi-step variant filtering and “rescue” strategy to accurately identify somatic mutations in PDX tumors. **a** Overview of the PDX variant calling workflow for engrafted tumors in the absence of paired-normal tumor samples and in the presence of mouse stroma. (see Methods for details). **b** Overview of the filter and rescue strategy used in the variant calling workflow for JAX PDXs. MTB: Mouse Tumor Biology Database, RD: Read depth, AF: Allele-frequency, FP: False positives, CKB: JAX Clinical Knowledgebase. **c** Proportion of mouse reads classified by Xenome for CTP and RNA sequencing data across all PDX models. **d** The recurrent frequencies of the mutated positions (after germline filtering) for various genes that were found to be recurrent in more than 25% of PDX samples. These were identified as additional false positive variants due to sequencing errors or mapping issues

A graphical overview of the workflow for calling somatic mutations and indels in PDX tumors is provided in Fig. 2a and b.

#### Preprocessing and removal of mouse reads

**Fig. 3:**
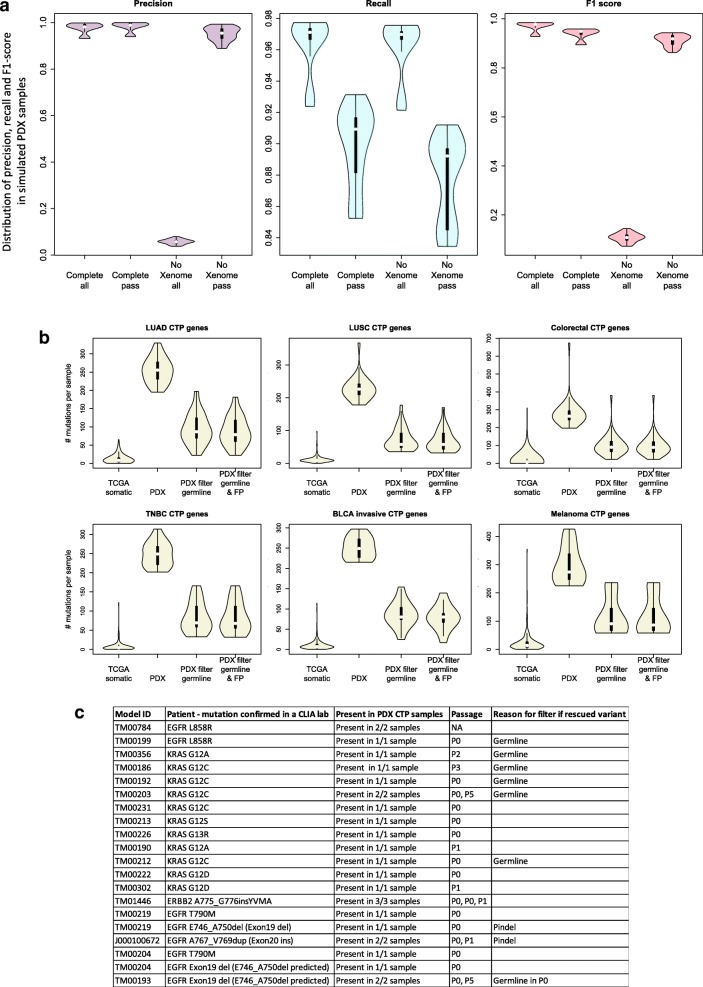
The PDX somatic mutation calling workflow improved the accuracy of predicting somatic mutations in engrafted tumors. **a** Benchmarking of the CTP variant calling workflow using 45 simulated sequencing datasets from different samples, sequence coverages, and mouse DNA content (Additional file 1: Table S2) using precision, recall and F1 score (see Methods) based on the input variants for each sample. Complete: variant calling pipeline with all steps included; NoXenome: variant calling pipeline with Xenome omitted; all: all variants called by the pipeline; pass: variants annotated as “PASS” in the pipeline which pass the hard filters, minimum read depth and minimum alternate allele frequency of the variant. **b** Distribution of mutational load per sample of non-silent coding somatic mutations for genes included on the CTP panel based on exome sequencing of TCGA samples and CTP-panel sequencing of PDX models. TCGA somatic: TCGA somatic mutations reported in maf files; PDX: all variants annotated as “PASS” (pass the hard filters, minimum read depth and minimum alternate allele frequency of the variant); PDX filter germline: all variants annotated as “PASS” and filtered from putative germline variants; PDX filter germline & FP: all variants annotated as “PASS” and filtered from putative germline variants and recurrent false positives. (LUAD: lung adenocarcinoma, LUSC: lung squamous cell carcinoma, Colorectal: colon and rectal cancer, TNBC: triple-negative breast cancer, BLCA invasive: muscle invasive bladder cancer). **c** Mutations identified in patient lung tumors that were retained in engrafted PDX tumors. Some of these variants were initially filtered out of the variant call analysis but subsequently reinstated using the variant rescue protocol (Additional file 1: Figure S2)

Human and mouse DNA reads were classified using Xenome [16], which has a reported performance of > 90% correct classification of mouse and human reads [69]. Only human reads were used for subsequent variant calling. The percentage of mouse reads identified in the engrafted tumors had a median value of 5.30% (range: 0.00163–65.1%; Fig. 2c). Using simulated sequencing datasets on the JAX Cancer Treatment Profile™ (CTP) panel [31], we verified that omitting the Xenome step for filtering the mouse reads resulted in many more FP variant calls (Fig. 3a; Additional file 2: Table S1). The FPs were due to the alignment of the mouse sequence reads to the human reference genome with mismatches that were subsequently called as variants with low-quality scores (QD). While the default thresholds for GATK hard filtering parameters [40] removed a large proportion of the FPs, applying Xenome to filter for human reads yielded higher precision and improvement in recall. Also, Xenome filtering maintained the correlation between the predicted versus actual allele frequencies, which would otherwise decrease with higher mouse contamination (Additional file 1: Table S2).

#### Filtering germline variants

Based on range of allele frequencies identified in sequences of normal human blood samples (Additional file 1: Text S1), the variants in each PDX tumor with an allele frequency of 40–60% or > 90%, and present in either public germline databases or our list of putative germline variants (Additional file 1: Table S3), were filtered out as germline variants. This was a more conservative approach given that these known germline variants in regions of copy number alterations where the ratio of both alleles was not balanced would not be filtered. Figure 3b shows that the germline filters reduced the estimated somatic mutational load in the PDX tumors (Additional file 1: Table S5) by about four-fold (Additional file 1: Table S4).

#### Filtering false positives due to systematic errors

Putative somatic mutations with no known effects in cancer that recur across large numbers of PDX samples are potential FPs arising from reference human genome assembly errors, sequencing errors, or alignment errors in low mappability regions [70]. To detect these putative FPs, we filtered out the variants at loci that were recurrently mutated in ≥25% (see Methods) of PDX tumors (Fig. 2d). The distribution of tumor types for each of these recurrently mutated positions (*n* = 52) was highly similar to the overall distribution of tumor types in the PDX resource with Pearson correlation coefficient > 0.9 (Additional file 1: Figure S2). This implies that these mutations were systematic errors and were not explicitly selected for in any tumor type, and thus, likely do not contribute to tumor biology or treatment response. While there was a negligible reduction in the overall mutational load after filtering highly recurrent variants, the filtering impact was notable for several known cancer-related genes (e.g., *ERBB4* and *MUC16*) (Fig. 2d, Fig. 3b and Additional file 1: Table S4).

#### Rescuing false negative variants

To address the balance of false positive and false negative mutation calls, we “rescued” single nucleotide variants and indels that were initially filtered out as germline using curated annotations available in the JAX Clinical Knowledgebase (CKB, https://ckbhome.jax.org/) [46, 47] (Additional file 1: Text S1, Additional file 1: Table S4). Overall, 127 unique variants from 52 genes (1.03% of the total and 2.21% of the filtered unique variants detected by the CTP platform) were rescued from 381 PDX tumors. Nine of these mutations were experimentally validated to be present in the PDX model (Fig. 3c). Almost all were initially filtered as germline events, as many well-known actionable cancer mutations (e.g., *BRAF* V600E and *KRAS* G12C) are present in the dbSNP database and occurred at frequencies that fell within our exclusion criteria. Two *EGFR* indels that were not called by GATK initially, but that were detected by Pindel were rescued as they were annotated clinically relevant.

### PDX somatic mutation workflow benchmarking

The benchmark testing of our somatic mutation workflow on a simulated dataset demonstrated high F1 score in variant calling, with high precision and an insignificant compromise on the recall (Fig. 3a, Additional file 2: Table S1 and Text S5). We observed that the allele frequencies of the true positive (TP) variants correlate well (Pearson correlation coefficient > 0.99) with the input allele frequencies for all samples (Additional file 1: Figure S3 and Table S2). Although the estimated allele frequencies were lower than the true allele frequencies, this difference was marginal and could be attributed to the reads carrying the variants being classified as non-human reads by Xenome, or not mapped to the genome. Moreover, all (20 out of 20) clinically relevant mutations experimentally validated or clinically reported in the corresponding patient tumors were detected in the PDX tumors (Fig. 3c).

### Gene expression analysis in PDXs

**Fig. 4:**
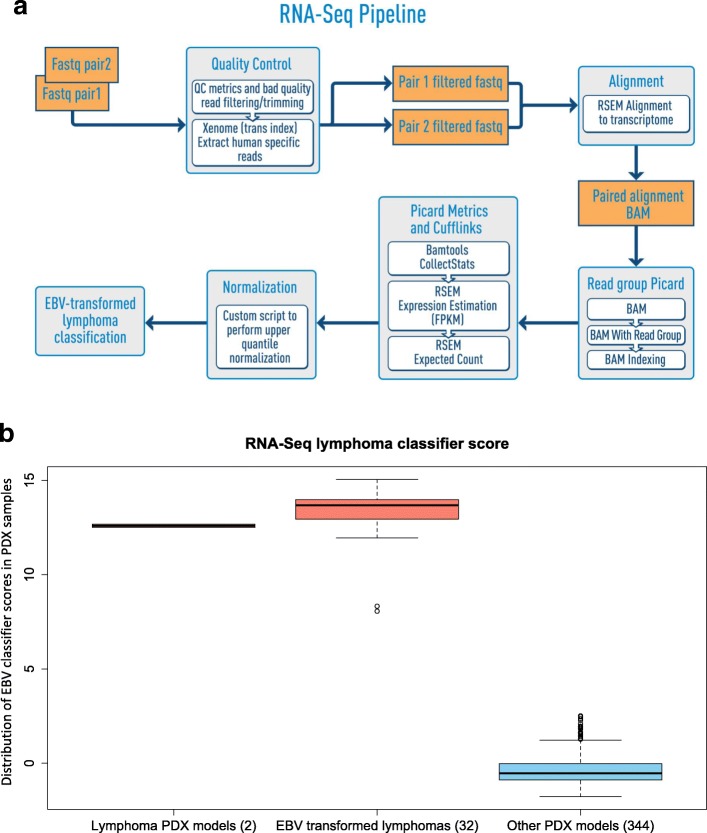
Expression profiling and identification of EBV-associated lymphomas from PDX RNA sequencing data. **a** Overview of the PDX RNA-Seq workflow (see Methods for details). **b** Distribution of lymphoma classification scores of PDX tumors

A graphical overview of the workflow for gene expression analysis of PDX tumors is provided in Fig. 4a.

#### Identifying EBV-associated PDX lymphomas by RNA-Seq expression data

Immunohistochemistry (IHC) for human CD45 is the primary screen for EBV-associated lymphomas in the JAX PDX Resource and is performed as one component of routine Quality Control procedures (see http://tumor.informatics.jax.org/mtbwi/pdxSearch.do). However, we also observed that EBV-associated lymphoma tumors display a distinct and highly reproducible expression pattern (Additional file 1: Text S2 and Figure S5). We implemented a 48-gene expression signature based on the most differentially expressed genes between EBV-associated lymphomas and non-EBV-associated tumors (Additional file 1: Table S6). This classifier was able to effectively distinguish PDX tumors that were either EBV-transformed from non-lymphoma PDX tumors or originated from human lymphomas (Fig. 4b). Overall, 8.5% (32 out of 376) of the non-lymphoma PDX samples with RNA-Seq data in the PDX resource progressed to EBV-associated lymphomas.

### Copy number alterations (CNA) analysis in PDXs

**Fig. 5:**
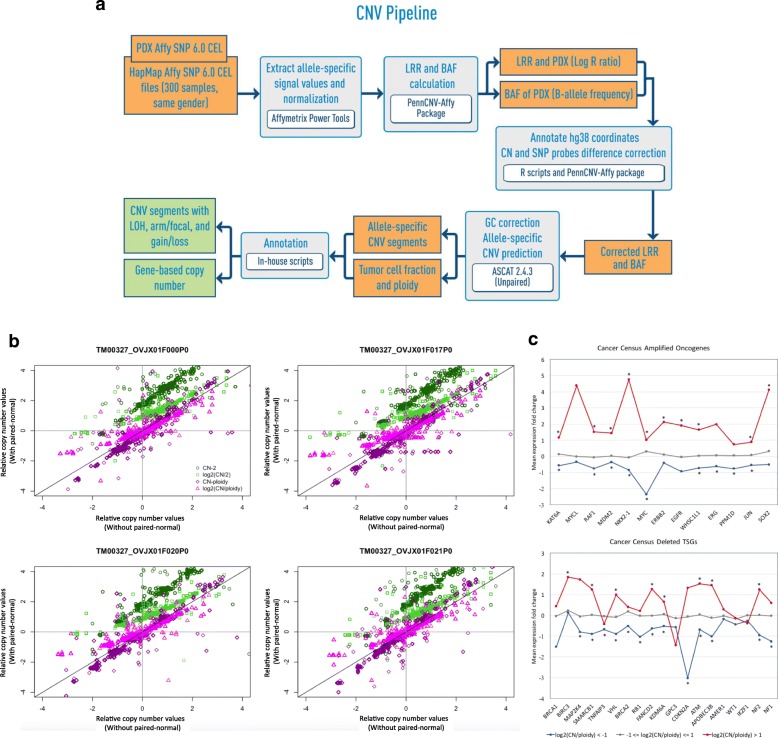
Somatic copy number gain and loss profiling from PDX SNP array data. **a** Overview of the copy number alteration and loss of heterozygosity prediction workflow for SNP array data from PDX tumors (see Methods for details). **b** Comparison of copy number for ovarian cancer PDX TM00327 relative to the estimated overall cancer genome ploidy of the PDX sample or the diploid state between analyses with and without paired-normal. The log_2_(CN/ploidy) gives the best agreement for comparing copy number analyzed with and without paired-normal. (CN-2: copy number difference relative to diploid state; CN/2: copy number ratio relative to diploid state, CN-ploidy: copy number difference relative to overall cancer genome ploidy, CN/ploidy: copy number ratio relative to overall cancer genome ploidy). c Mean expression fold change of genes with normal copy number, copy number gain (log_2_(CN/ploidy) > 1) and copy number loss (log_2_(CN/ploidy) < − 1) state for a selected list of known oncogenes that are amplified in cancers and for known tumor suppressor genes that are deleted in cancers from the Cancer Gene Census [57]. Over-expressed and under-expressed genes marked with * indicates significant differences in expression fold change with copy number gain or loss state respectively relative to the normal state across all PDX samples

A graphical overview of the workflow for calling copy number alterations in PDX tumors is provided in Fig. 5a.

#### Effect of mouse DNA on CNA calls

We studied the effect of mouse contamination on array data by hybridizing DNA of the PDX host strain, NOD.Cg-*Prkdc*^*scid*^*Il2rg*^*tm1Wjl*^/SzJ (aka, NSG) mouse strain from The Jackson Laboratory (strain 005557), on the human SNP array. The signal intensity from mouse DNA was negligible (Additional file 1: Figure S6). Samples with higher mouse content were more likely to fail the standard array quality control, due to the lower amount of human DNA to give sufficient probe signal, thus enabling samples with substantial mouse contamination to be screened out.

#### Absence of matched non-tumor to call somatic copy number alterations

We developed a single-tumor CNA analysis method, by normalizing the SNP array signal intensity of the tumor sample with that of HapMap [55, 71] samples of the same sex. For the PDX samples with paired normal samples (*n* = 9), we observed the overall high similarity between the segmented copy number profiles analyzed with and without the paired-normal sample (Additional file 1: Figure S7). The gene-based log2(total CN/ploidy) showed good correlation between the single-tumor and tumor-normal CNA analysis (Pearson correlation > 0.81), with 8 out of 9 PDX samples correlating > 0.93 (Fig. 5a and Additional file 1: Table S7), indicating that the single-tumor CNA analysis was robust.

#### Establishing the appropriate baseline to call copy number gains and losses

Due to aneuploidy in cancer genomes, we analyzed the effects of using different “normal” states to compute copy number gains and losses using a list of significantly amplified and deleted genes from TCGA (Additional file 1: Fig. S8, Additional file 2: Table S13). When the overall cancer genome ploidy was used as the baseline, we observed that a larger proportion of the significantly amplified and deleted genes were called as copy number gains and losses among the PDX samples respectively (Additional file 1: Figure S9). However, a large fraction of both significantly amplified and deleted genes were classified as amplified when copy number alterations were calculated relative to the diploid state, indicating that calling copy number changes relative to the diploid state could classify gain and loss genes incorrectly in the PDX samples. While the average ploidy might not be estimated consistently across the tumor samples for the same model, the copy number changes relative to overall cancer genome ploidy remained consistent (Fig. 5b and Additional file 1: Figure S7).

#### Effects of copy number alterations on expression changes

We observed that the estimated copy number gains and losses of known oncogenes (*n* = 23) and tumor suppressor genes (*n* = 40) [57], relative to the average ploidy per PDX sample, generally results in expression changes in the same direction as the copy number change (Additional file 1: Table S8) [12, 72, 73]. In a subset of PDX samples for which both expression and copy number data were available (*n* = 194), the over and under-expression were computed relative to the “normal” expression of each gene estimated by the average expression in samples with normal copy number state. While this approach did not account for all mechanisms of gene regulation, we were able to better estimate the normal expression for genes compared to using the mean expression (z-score) across all tumor types, which could be biased for frequently aberrated genes such as *MYC*, which tends to be frequently amplified and over-expressed across many tumor types [74, 75]. Most of these genes show significant over-expression with copy number gain and significant under-expression with copy number loss across the PDX samples (*p* < 0.05) (Fig. 5c, Additional file 1: Figure S10 and Text S3). These results support using overall cancer genome ploidy as the baseline to call copy number gain and loss.

### Comparison of genomic and transcriptomic profiles of PDX models and TCGA patient tumors

Because we lacked paired-normal samples for most models in the JAX PDX resource, we were unable to experimentally validate the somatic mutations predicted by our workflows. As an alternative approach to validation, we compared the genomic and transcriptomic profiles for the JAX models to data for the same tumor type available from TCGA and assessed the overall concordance of patterns in mutation frequency, gene expression, and copy number alterations.

#### Frequently mutated genes in primary patient tumors in TCGA are detected in the PDX resource

**Fig. 6:**
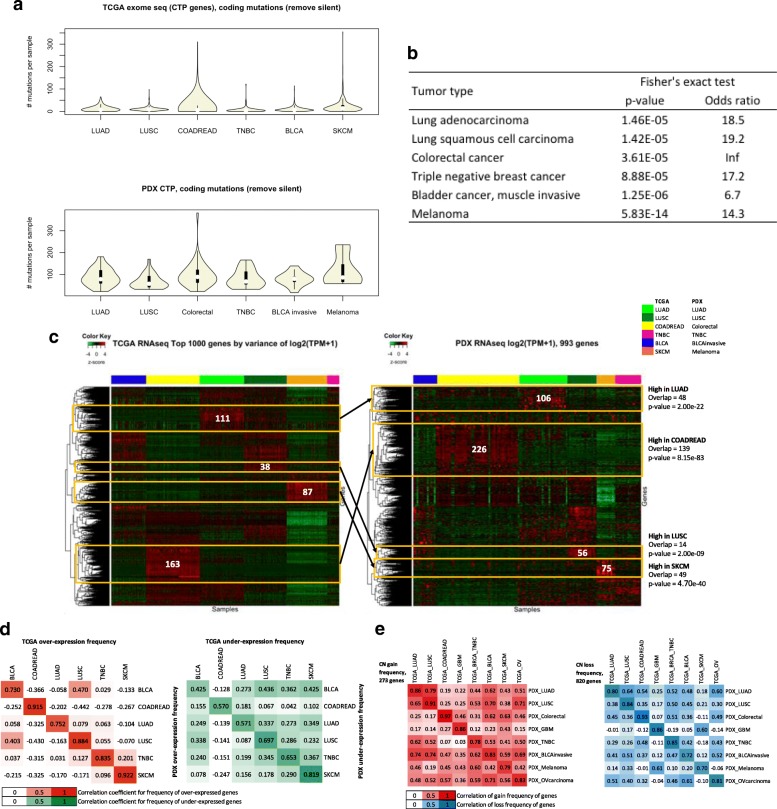
Comparison of somatic genomic and transcriptomic profiles between JAX PDX resource and TCGA tumor cohorts. **a** Distribution of mutational load of non-silent coding somatic mutations for genes on the CTP panel based on exome sequence data for TCGA samples and CTP panel-based sequence data for JAX PDX models (all filters included). (LUAD: lung adenocarcinoma, LUSC: lung squamous cell carcinoma, COADREAD: colorectal adenocarcinoma, Colorectal: colon and rectal cancer, TNBC: triple-negative breast cancer, BLCA: urothelial bladder carcinoma, BLCA invasive: muscle invasive bladder cancer, SKCM: skin cutaneous melanoma, GBM: glioblastoma multiforme). **b** Overlap of CTP panel genes that have non-silent coding somatic mutations with > 5% mutation frequency in TCGA data with genes that have at least one non-silent coding somatic mutation in PDX CTP data (all filters and rescue of clinically relevant variants included) for each tumor type. Fisher’s exact test (Additional file 1: Table S9) was used to compute the significance of the overlap. **c** Hierarchical clustering of z-score of expression (log_2_(TPM + 1)) of top 1000 most varying genes of TCGA RNA-Seq samples across different tumor types. The same set of genes (omitting non-expressed genes) was used as input for unsupervised hierarchical clustering of PDX models for all tumor types represented in the JAX resource. Gene sets identified as having high expression in specific tumor types had significant overlap between TCGA and PDX samples. **d** Correlation between PDX models and TCGA samples of over-expressed (z-score of log_2_(TPM + 1) > 1, green) or under-expressed (z-score of log_2_(TPM + 1) < − 1, orange) genes across multiple tumor types. **e** Correlation between PDX and TCGA tumors for the frequency of copy number gain (red) or loss (blue) of selected genes frequently amplified or deleted in TCGA tumors as predicted by GISTIC

The distribution of somatic coding non-silent mutational load of the CTP genes for each tumor type was comparable between PDX and TCGA (Fig. 6a). Despite the much smaller sample size for each PDX tumor type, we observed a higher mutational load in colorectal cancer and melanoma among other tumor types, which is consistent with TCGA. Given that there were more samples in the TCGA cohorts, we compared the genes that were mutated at 5% frequency with genes that were mutated in at least one sample within the same tumor type in the PDX resource. Almost all genes mutated at high frequencies in TCGA tumors were mutated in PDX tumors, with significant *p*-values (*p* < 1 × 10^− 4^) by Fisher’s exact test (Fig. 6b, Additional file 1: Table S9). These results indicate that the key drivers by mutation within each cancer type are preserved in PDX tumors.

#### Expression signatures of primary patient tumors in TCGA are recapitulated in the PDX resource

The top 1000 most varying genes across 6 TCGA tumor types measured by the variance of expression z-scores (Additional file 1: Table S10) were able to independently cluster both TCGA samples and the PDX samples by their tumor types (Fig. 6c). We observed that clusters of genes that were highly expressed in specific tumor types in TCGA were recapitulated in the PDX expression data (hypergeometric p-value < 1 × 10^− 8^), which demonstrated the replicability of TCGA expression signatures in the PDX resource. The frequencies of over- and under-expression for the top-varying genes for each tumor type displayed better correlation for the same tumor type for PDX versus TCGA compared to other tumor types (Fig. 6d). The varying level of concordance between different tumor types in TCGA data was also maintained in the PDX versus TCGA comparison (Additional file 1: Figure S11). Alternatively, the differentially expressed genes of each tumor type versus all other tumors within the TCGA or PDX samples displayed significant overlaps (p < 1 × e^− 6^), despite different sample sizes and the different proportion of tumor types (Additional file 1: Table S11).

#### Copy number profiles of primary patient tumors in TCGA are recapitulated in the PDX resource

The frequency of genome-wide copy number alterations for each tumor type in the PDX resource (Additional file 1: Table S12 and Fig. S12A) was similar to the primary tumors in TCGA (Additional file 1: Fig. S12B). Moreover, the PDX tumors had the highest correlation in gain and loss frequencies of significantly amplified and deleted genes for the same tumor type in TCGA compared to other tumor types (Fig. 6e and Additional file 1: Figure S13A). The varying levels of correlation between different tumor types were preserved between the TCGA versus TCGA tumors and the TCGA versus PDX tumors (Fig. 6e and Additional file 1: Fig. S14). Consistent with the earlier observations, there was a weaker concordance with TCGA data when amplification and deletion were called relative to the diploid state (Additional file 1: Figure S13B).

## Discussion

Genomic data analysis workflows designed to call somatic mutations (SNVs, indels), copy number alterations and gene expression from PDX sequencing or array data require balancing sensitivity and specificity [25, 70], especially when paired normal samples for engrafted tumors are not available. Using genomic and transcriptomic data from models in the JAX PDX resource, we developed and tailored data analysis workflows to reliably identify true somatic mutations, copy number alterations, and expression changes using genomic and transcriptomic data from PDX tumors that lack paired non-tumor samples. Key elements of our analysis guidelines and recommendations are summarized below.

We recommend using multiple data sources for germline variants and cancer relevant variants to fine-tune workflows for calling somatic mutations to achieve a reasonable balance of false-negative and false positives. Variant calling pipelines should be re-run periodically as new data about germline and cancer-relevant variants become available. Our major recommendations for data analysis workflows for calling somatic mutations for PDX tumors in the absence of paired-normal samples include the following:

- Remove mouse reads with Xenome (or equivalent) [16, 69, 76, 77] to eliminate variants called from mouse reads that map to highly conserved regions of the human reference genome.
- Optimize quality thresholds (variant read depth and allele frequencies, and various sequencing and alignment quality values) to filter for high quality variant calls for any given capture panel and sequencing coverage.
- Adjust quality filters to detect low frequency variants (> 5%) with high confidence in order to detect clinically-relevant variants that are present in minor clones.
- Filter variant calls to remove those that are likely germline variants [43–45] to improve somatic mutation calling.
- Filter highly recurrent (> maximum somatic mutation frequency) mutations to remove false positives arising from sequencing or analysis-related errors.
- Reinstate (rescue) variants with putative clinical relevance [46, 78–81] that meet quality thresholds but are initially filtered out as germline or highly recurrent. This “variant rescue” process will likely also reclaim germline variants that are associated with cancer susceptibility and treatment response [82, 83] which may be important for selecting PDX models for dosing studies.

We recommend using publicly available data sets to generate a proxy for sex-matched normal samples in order to estimate copy number alterations in an engrafted PDX tumors. Our major recommendations for data analysis workflows for estimating copy number alterations by SNP arrays for PDX tumors in the absence of paired-normal samples include the following:

- Normalize SNP array signal intensity with a large number of normal samples [55, 71] that correspond to the sex of the patient associated with the PDX model.
- Estimate copy number gains and losses using copy number ratio relative to overall cancer genome ploidy

Although not evaluated for the work reported here, data analysis methods have been developed by other research groups to estimate copy number alterations in absence of paired-normal samples from whole-exome or whole-genome sequence data [84–86]. Comparison of these methods to CNA evaluation by SNP arrays will require additional genome characterization for the PDX tumors in the JAX repository.

We recommend using gene expression data from engrafted tumors for both quality assurance of PDX models in addition to comparing gene expression levels. Our major recommendations for data analysis workflows for comparing gene expression levels for PDX tumors in the absence of paired-tissue RNA include the following:

- Screen for EBV-transformed lymphomas using lymphoma expression classifier score in addition to a primary screen of paraffin embedded tumor samples using immunohistochemistry for human CD45+ cells.
- Use mean expression across samples of all tumor types (z-score) or average expression across samples with “normal” copy number state for comparison of gene expression levels.

The availability of RNA-Seq data for engrafted tumors provides an opportunity for predicting fusion genes and various software tools are available for this purpose [87, 88]. Benchmarking fusion gene prediction methods for engrafted tumors is planned for a future extension of our PDX analysis workflows.

To assess the quality of our genomic characterization workflows, we compared the results of our workflows for PDX tumors with data from TCGA. Overall, our analysis results demonstrated that patterns of frequently mutated genes, copy number alterations, and gene expression were comparable in PDX and TCGA samples of the same tumor type. Using colorectal cancer as an example, we demonstrated that pathways known to be perturbed in this cancer were altered consistently in PDX and TCGA tumors [58] (Additional file 1: Text S6 and Fig. S15), with similar combinations of alterations occurring at comparable frequencies.

One persistent difference in PDX and TCGA samples was in the predicted mutational load of comparable tumors. Despite implementing multiple filters to remove putative germline and other FP mutations, the mutation rates calculated for the JAX PDX tumors are higher than data for corresponding tumors from the TCGA (Additional file 1: Text S4). This could be because PDX tumors were sequenced at a higher coverage compared to TCGA tumors and/or because germline variants were not completely filtered out in the PDX samples. Another possible reason for this difference that many of the human tumor samples used to generate PDX models were from metastatic lesions and patients with prior treatment whereas many of the tumor samples in TCGA represent early stage tumors which are treatment naive. Overall, 51.0 and 31.4% of the PDX models in the JAX resource are late-stage and high-grade tumors respectively, while 32.1% of TCGA tumors [89] (Additional file 1: Table S5) are late-stage tumors. These PDX tumors are thus expected to harbor more mutations due to tumor evolution [90, 91]. Further, previous studies have noted that PDX engraftment success is higher for late-stage tumors that are likely to have more aggressive phenotypes than for early-stage tumors [92, 93]. As such, it is possible tumors from PDXs harbor more mutations due to a bias in engraftment success.

## Conclusions

In conclusion, the bioinformatics analysis workflows and guidelines (https://github.com/TheJacksonLaboratory/PDX-Analysis-Workflows) that we have developed for the analysis of genomic data generated from PDX tumors lacking paired non-tumor tissue result in accurate detection of somatic alterations in PDX models. We demonstrate the effectiveness of our workflows by validating with simulated data. In addition, we show that there is high concordance of the genomic and transcriptomic profiles of the PDX models in the JAX PDX resource with relevant tumor types from The Cancer Genome Atlas (TCGA).

## Additional files

Additional file 1: Supplementary Texts S1-S6, Supplementary Figures S1-S15, Supplementary Tables S2-S12. (DOCX 40439 kb)

Additional file 2: Supplementary Tables S1, S13 and S14. (XLSX 94 kb)

## Data Availability

Summarized genomic data for the models in the JAX PDX resource can be accessed from the PDX portal hosted by the Mouse Tumor Biology (MTB) Database (http://tumor.informatics.jax.org/mtbwi/pdxSearch.do). Access to raw data is provided upon request. The implementation of the bioinformatics analysis workflows and guidelines described here are available in https://github.com/TheJacksonLaboratory/PDX-Analysis-Workflows.

## Abbreviations

CKB: Clinical Knowledgebase
CNA: Copy number alterations
CTP: Cancer Treatment Profile
EBV: Epstein-Barr virus
FP: False positive
Indels: Insertions and deletions
JAX: The Jackson Laboratory
PDX: Patient-derived xenograft
SNV: Single nucleotide variations
TCGA: The Cancer Genome Atlas
TP: True positive
