# Supplementary material for: Genomic data analysis workflows for tumors from patient-derived xenografts (PDXs): challenges and guidelines
Source: BMC Med Genomics. 2019 Jul 1;12:92. doi: 10.1186/s12920-019-0551-2 (PMC6604205; doi:10.1186/s12920-019-0551-2)
Supplement: Supplementary file 1 — Supplementary Texts S1-S6, Supplementary Figures S1-S15, Supplementary Tables S2-S12. (DOCX 40439 kb) [file 12920_2019_551_MOESM1_ESM.docx]

**Additional File 1**

**Genomic data analysis workflows for tumors from Patient-Derived Xenografts (PDXs): challenges and guidelines**

Xing Yi Woo^#,1^, Anuj Srivastava^#,1^, Joel H. Graber^#,3^, Vinod Yadav^1,5^, Vishal Kumar Sarsani^2,4^, Al Simons^2^, Glen Beane^2^, Stephen Grubb^2^, Guruprasad Ananda^1^, Rangjiao Liu^1,6^, Grace Stafford^2^, Jeffrey H. Chuang^1^, Susan D. Airhart^2^, R. Krishna Murthy Karuturi^1^, Joshy George^*,1^, Carol J. Bult^*,2^

^1^ The Jackson Laboratory for Genomic Medicine, Farmington, CT 06030, USA

^2^ The Jackson Laboratory for Mammalian Genetics, Bar Harbor, ME 04609, USA

^3^ MDI Biological Laboratory, Bar Harbor, ME 04609, USA

^4^ Current affiliation: University of Massachusetts, Amherst, MA 01003, USA

^5^ Current affiliation: Massachusetts Institute of Technology, Cambridge, MA 02139, USA

^6^ Current affiliation: Novogene Corporation, Rockville, MD 20850, USA

^#^ The authors contributed equally to this work.

^*^ Corresponding authors: Carol J. Bult (Email: Carol.Bult@jax.org), Joshy George (Email: Joshy.George@jax.org)

**Text S1: Analysis of germline variants**

To enhance filtering out germline variants from somatic mutations, we sequenced and analyzed 20 normal blood samples using the CTP-targeted panel. These normal blood samples included 8 HapMap individuals, and 12 other blood samples collected from cancer patients in the JAX PDX and Clinical Genomics resource (3 glioblastoma multiforme, 1 neuroblastoma, 1 brain teratoma, 1 renal cell carcinoma, 6 angiosarcoma). As shown in Figure S1A and S1B, 87% of the variants identified in normal blood had allele frequencies of 40% - 60%, or >90% across all the samples, indicating the presence of heterozygous or homozygous common variants, respectively. 91% of the variants identified in these 20 samples were annotated in the public germline databases. 4% of these variants were not found in public germline databases, but were recurrent in these normal samples or across the PDX tumors in our collection (Figure S1C) and thus were added to our list of putative germline variants. Only 5% of all of the variants in the 20 samples were private events.

We observed that 46% of the variants in the normal samples are present in the COSMIC database (Figure S1A). The germline filters might filter out actual somatic events in each PDX sample, leading to false negatives. However, retaining all variants represented in cancer variant databases such as COSMIC would lead to excess false positives.

**Text S2: Expression analysis of EBV-associated lymphomas**

The PDX tumors identified as EBV-associated routinely showed higher correlation in expression profiles than distinct pairs of PDX models derived from common original tumor materials, regardless of the platform in which the expression was measured (RNA-Seq, Affymetrix Human Gene 1.0 ST arrays or Human Gene 133 Version 2 arrays) (Figure S5). This expression profile was also independent of the tissue of origin of the tumors from which the EBV-associated lymphomas were derived. Given the high similarity in expression profiles, we identified a gene signature based on the highest over- and under-expressed genes of the EBV-associated lymphomas relative to the non-EBV-associated tumors (data not shown). Using gene set analysis, we observed that genes associated with B-lymphocytes and other immune processes were over-expressed, while cell-to-cell communication and adherence genes were suppressed (data not shown).

**Text S3: Analysis of copy number alterations with expression changes**

The phenomenon of copy number gain and loss resulting in over and under expression of genes did not hold when we performed a global analysis across all genes rather than on selected oncogenes and tumor suppressor genes. We speculate this is because not all genes are expressed in all PDX tissue types even though they are in regions affected by copy number alterations. Furthermore, the expression of many genes, despite being non-altered regions, might have been regulated by other mutations or epigenetic mechanisms in the tumors.

**Text S4: Higher mutational load in PDX tumors compared to TCGA tumors**

Our observation of a higher mutational load in PDX tumors relative to corresponding tumor types in TCGA could be due to the fact that the PDX tumors were sequenced at a higher coverage (>900X) using the CTP-targeted panel, and thus more variants were detected per base pair compared to exome sequencing (~100X) of TCGA tumors. Moreover, germline variants were not completely filtered in the PDX samples. These include known germline variants with allele frequency outside the range of 40% - 60% and >90%, possibly due to errors in allele frequency estimation or copy number alterations at the variant position, as well as private germline variants.

**Text S5: Mapping to alternate loci in the genome assembly**

The GRCh38.p5 human genome assembly includes 262 regions of alternate assemblies (ALT regions) to account for human chromosomal regions that exhibit sufficient variability to prevent adequate representation by a single sequence^1^. As such, we aligned the reads to both primary and alternate chromosomal reference sequences using BWA-MEM with ALT-aware alignment. When alignment is performed without ALT-aware functionality, the recall of the variants is much lower (~30%) than our standard workflow with or without hard-filtering (Figure S4 and Additional file 2: Table S1). The correlation of allele frequencies also decreases and the reduction in median allele frequency increases up to 15% (Table S2).

**Text S6: Altered genes in JAX PDX and TCGA colorectal tumors**

Figure S15 shows the frequency of genes altered PDX and TCGA tumors for each genomic datatype for colorectal cancer. These genes are identified by commonly affected pathways in colorectal cancer reported in TCGA studies^2^. For both PDX and TCGA cohorts of colorectal cancer, we observed high frequencies in the 1) mutation of *APC* and over-expression of *AXIN2* in the WNT signaling pathway, 2) amplification of *IRS2* in the PI3K signaling pathway, 3) copy number loss of SMAD2 and *SMAD4* in the TGF-β signaling pathway, 4) under-expression of *BRAF* in the RTK-RAS signaling pathway, and 5) copy number loss of *TP53* in the TP53 signaling pathway.

**References**

1 Jager, M. *et al.* Alternate-locus aware variant calling in whole genome sequencing. *Genome Med* **8**, 130, doi:10.1186/s13073-016-0383-z (2016).

2 Cancer Genome Atlas, N. Comprehensive molecular characterization of human colon and rectal cancer. *Nature* **487**, 330-337, doi:10.1038/nature11252 (2012).

**Figure S1.**

1. The annotation of unique variants (n=2656) generated by the JAX Cancer Therapy Profile (CTP) panel sequencing for 20 normal samples using public databases (dbSNP Build 144, 1000 Genomes, ExAC version 0.3, and COSMIC version 80) shows that the majority of the variants are known germline variants and that close to half have been annotated as somatic mutations in other cancer samples.
2. The allele frequencies of the variants fall mainly in 40% - 60% (heterozygous) and 90% - 100% (homozygous) across all normal samples.
3. A proportion of the unknown (not in public databases) germline variants are recurrent across the 20 normal samples.
4. Recurrent variants (present in more than 1 sample) found in 20 normal samples are also recurrent across 236 PDX models of different tumor types.

A


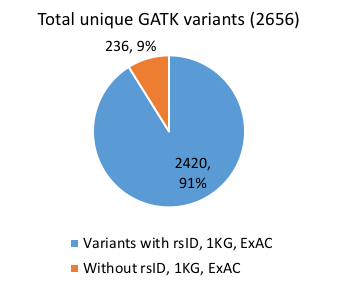

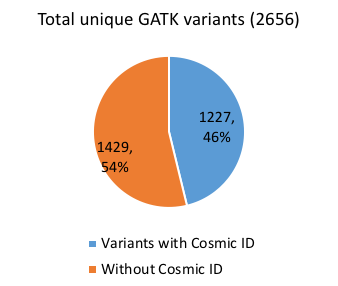


B


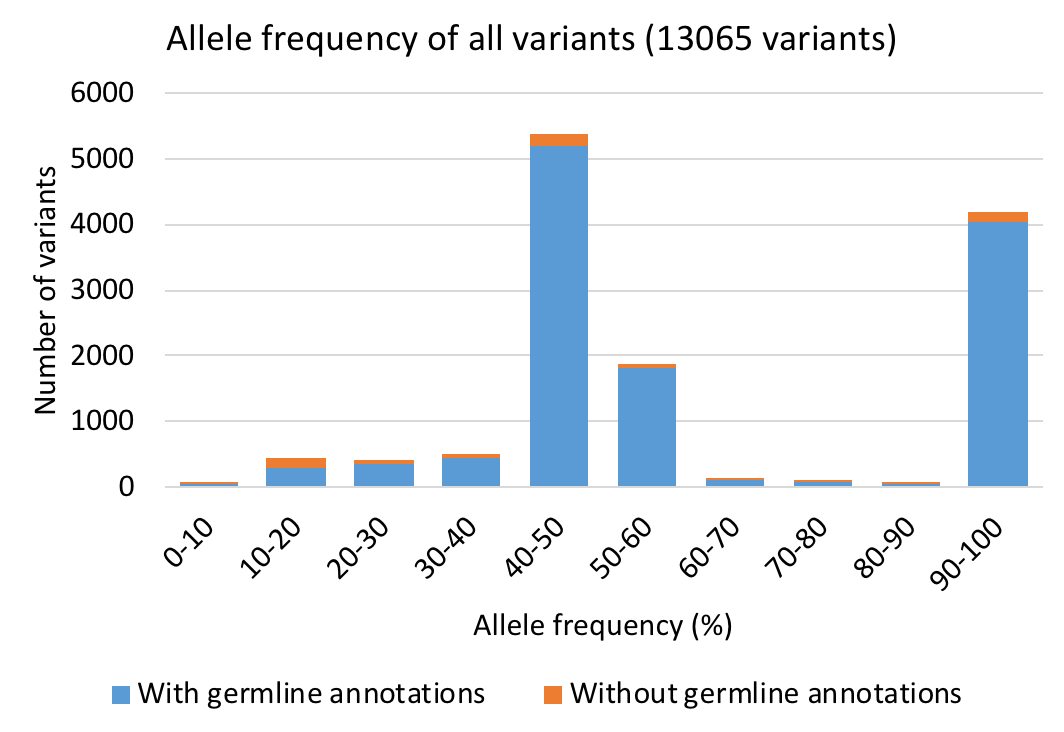


C


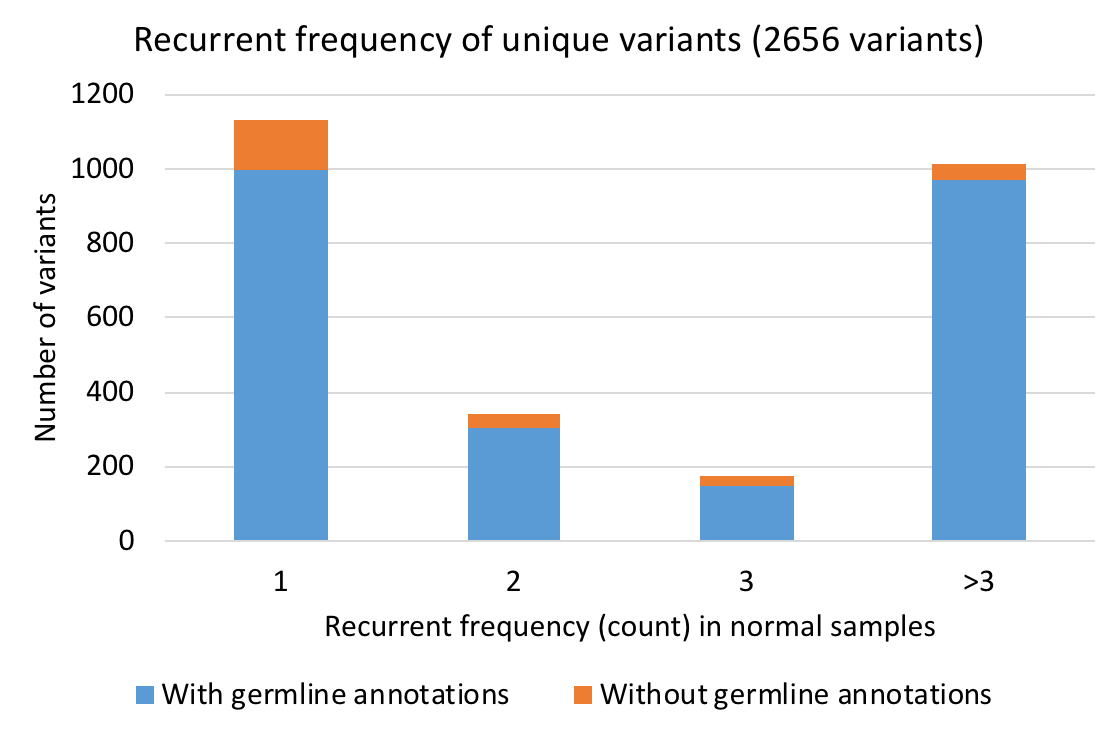


D


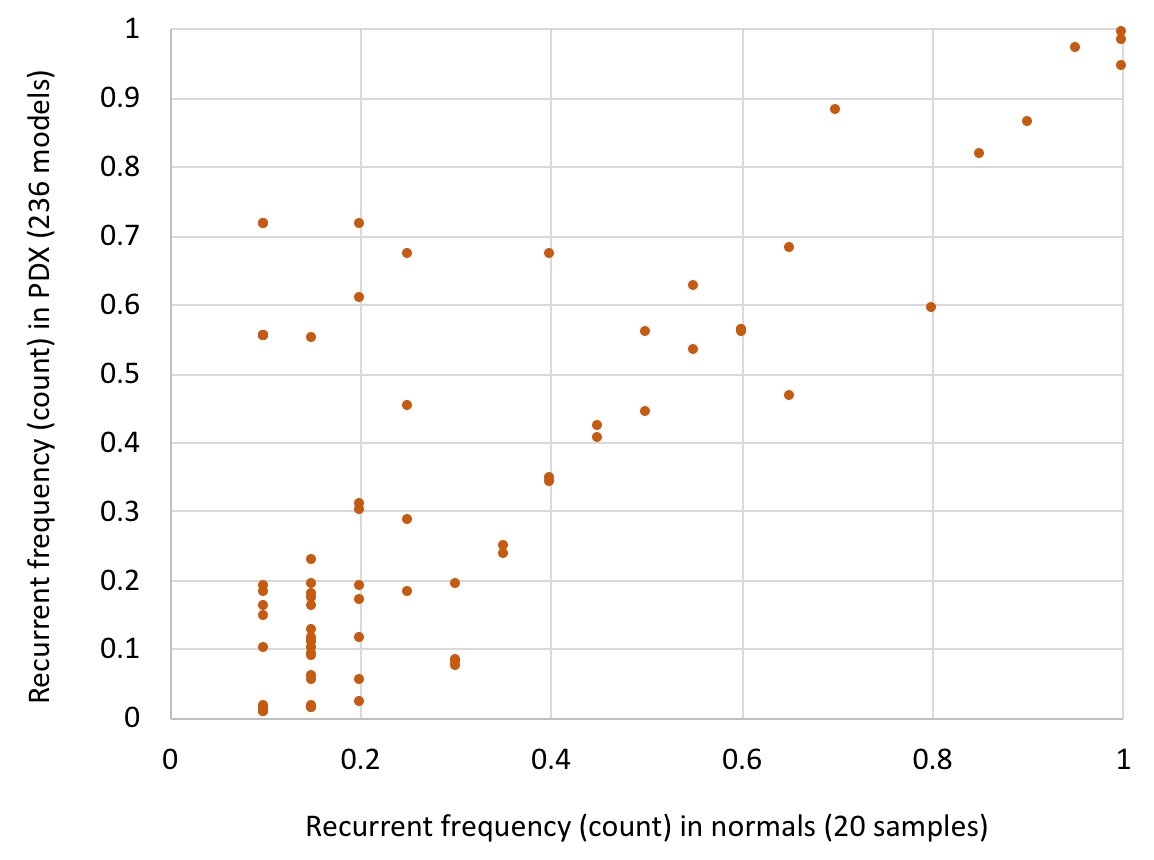


**Figure S2.**

1. The overall distribution of tumor types for 52 frequently mutated (i.e., >25% recurrence) sites across 236 JAX PDX models (dark blue) is similar to the distribution of frequently mutated sites normalized by the recurrent frequency of the mutation (illustrated by three typical cases in light grey).
2. The normalized tumor type frequencies (values of each bar in A) for each frequently mutated position (n=52, grey in A) are highly correlated with the overall tumor type frequencies for 236 PDX models (dark blue in A). The correlation coefficient is >0.9 but reduces slightly towards lower recurrent frequencies but maintains at >0.9 correlation at the threshold (>25%) of selecting false positive variants.

A

B


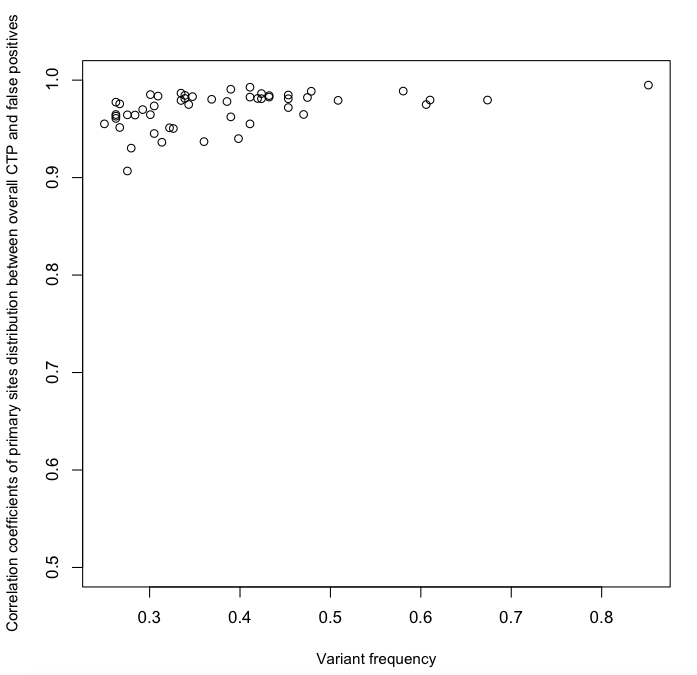


**Figure S3.**

(A) Plot showing the high correlation of alternate allele frequencies (ALT_AF) for input and true positive output variants called by the PDX variant calling workflow for one of the simulated samples. See Table S2 for the correlation coefficients for all simulated samples. [All=all variants called by the workflow; Pass= variants that meet GATK hard filter criteria as well as the minimum read depth and minimum alternate allele frequency criteria described in Methods].

(B) Box plot showing a small reduction in the alternate allele frequencies (ALT_AF) of the true positive output variants compared to the input variants for one of the simulated samples. See Table S2 for the median difference in ALT_AF for all simulated samples. [All=all variants called by the workflow; Pass= variants that meet GATK hard filter criteria as well as the minimum read depth and minimum alternate allele frequency criteria described in Methods].

A


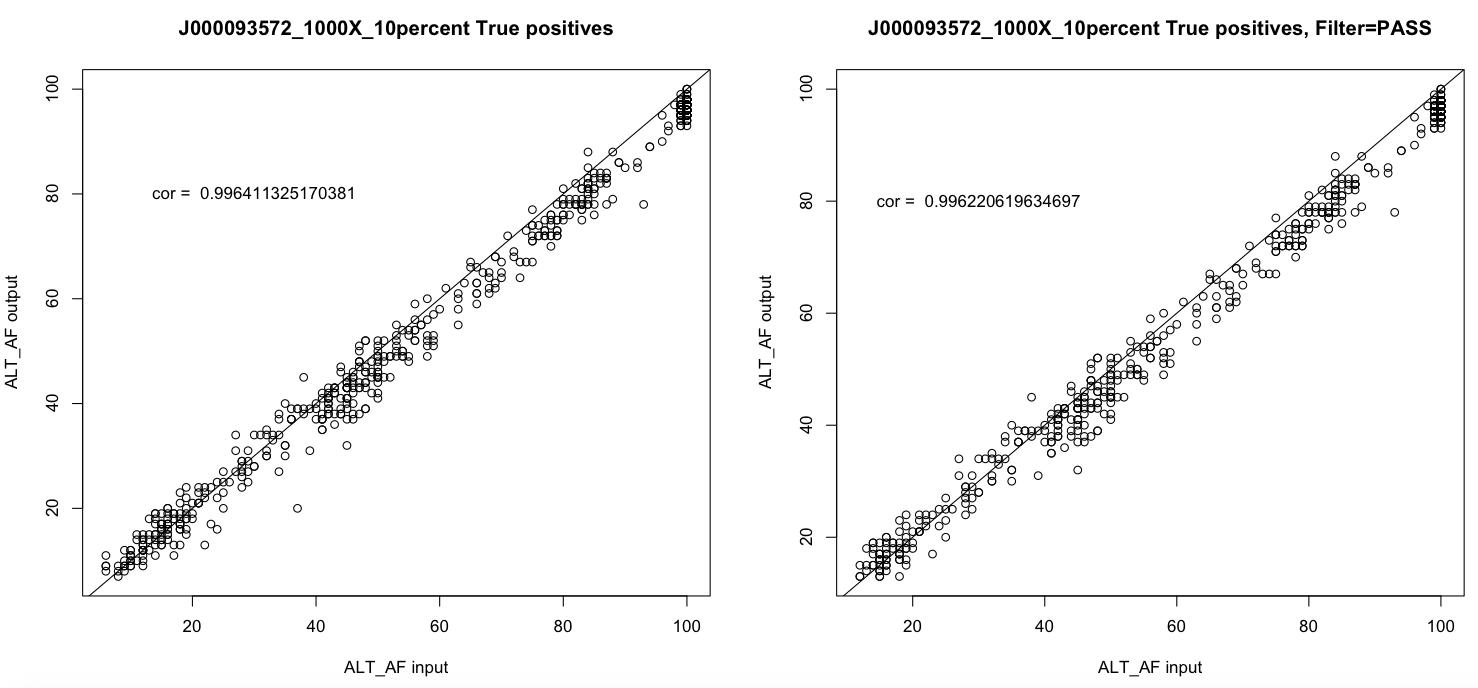


B


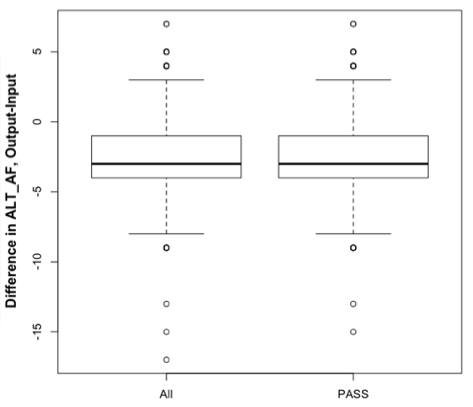


**Figure S4.**

Results of benchmarking the PDX Cancer Therapy Profile (CTP) variant calling workflow using 45 simulated sequencing datasets with different samples, sequencing coverages, and degrees of mouse DNA content (see Additional file 2: Table S1). The graphs show that scores for precision, recall and F1 are lower when Xenome and BWA with ALT-Aware are omitted from the workflow. [Complete= variant calling workflow with all steps included; NoXenome= variant calling workflow with Xenome omitted; NoAltaware= variant calling workflow using GRCh38 reference with alternate sequences but using standard BWA for mapping instead of BWA-ALT-Aware; All= all variants called by the workflow; Pass= variants that meet GATK hard filter criteria as well as the minimum read depth and minimum alternate allele frequency criteria described in Methods].


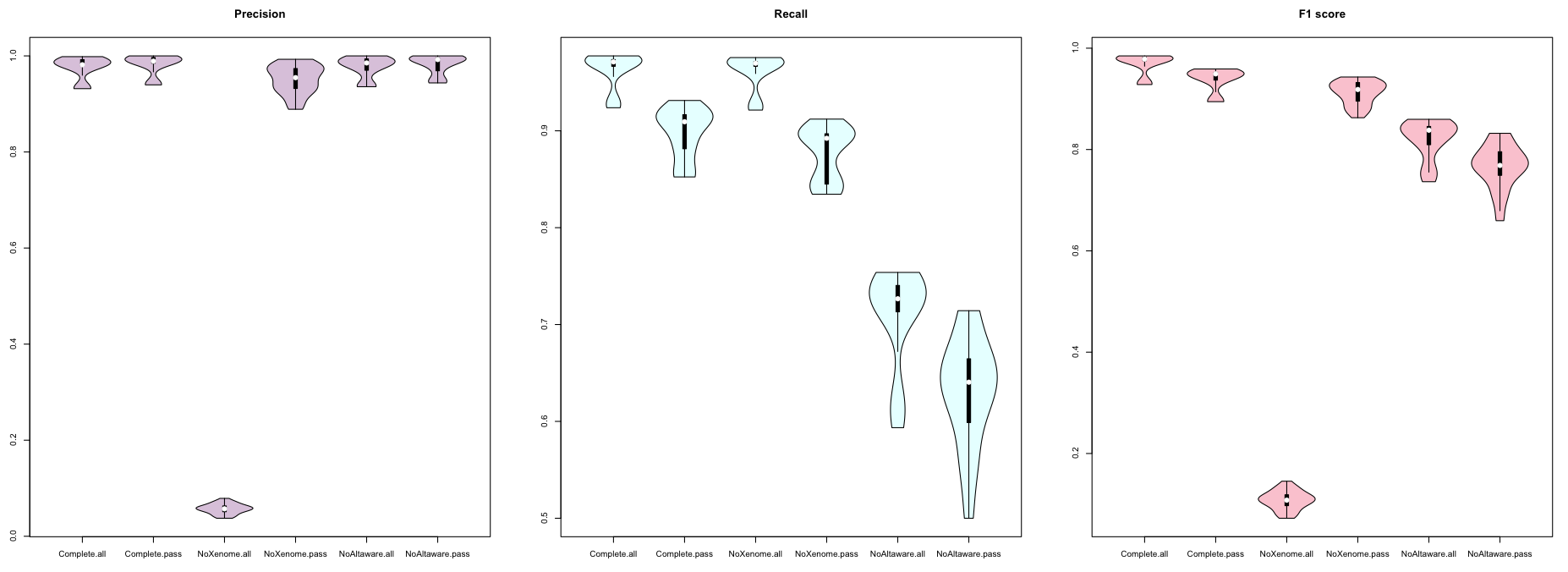


Distribution of precision, recall and F1-score

in simulated PDX samples

**Figure S5.**

1. Hierarchical clustering of correlations of gene expression between pairs of models. Red indicates perfect correlation and blue indicates perfect anti-correlation. The two largest red blocks (highlighted in white and yellow) show the mouse sequence contaminated and EBV transformed models. The other blocks, which much lower average correlation, typically show related tumor types (e.g., the lower right block is all neurological tumors).
2. A small fraction of tumors, highlighted in white in (A), that were heavily contaminated by mouse tissues were clustered with expression of NSG mouse skin sample (NSG_Skin_CTL).
3. EBV lymphoma models, highlight in yellow in (A), show an extremely highly correlated expression pattern with lymphoma models (sample names beginning with “LY”) regardless of the original tissue or tumor type.

A


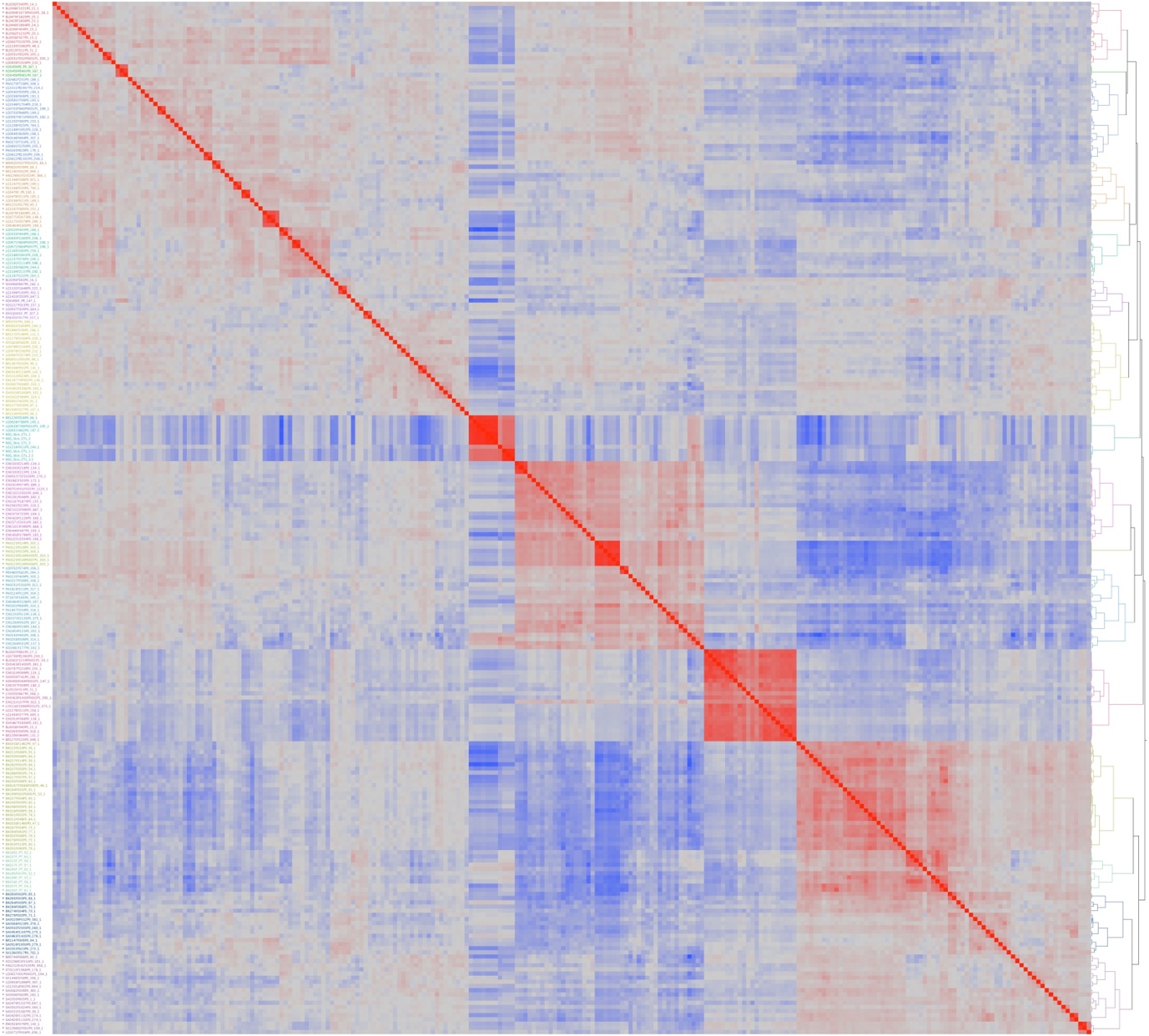


B C


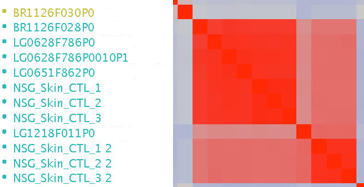

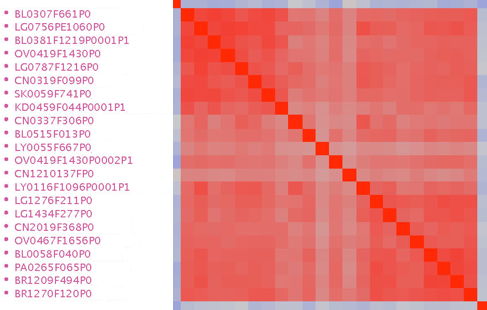


**Figure S6.**

1. The distribution of probe intensities of the SNP array for PDX samples with different human DNA content shows that the lack of human DNA in a sample results in overall low probe signal intensities. The percentage of human reads were given by Xenome for CTP sequencing as an estimation for human DNA content on the SNP array. [green= > 99% human DNA; red= < 50% human DNA with QC failure of SNP array CEL file; black= 100% NSG mouse DNA]
2. SNP arrays with lower human DNA content in PDX samples are more likely to result in QC failure [black squares=successful CNA prediction; orange circles=CEL file QC failure; red triangles= failure in ASCAT analysis].

A


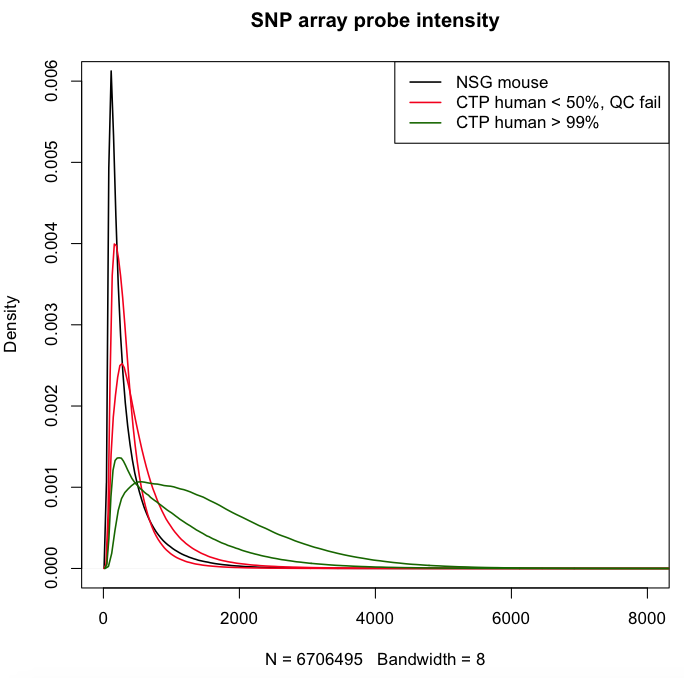


B


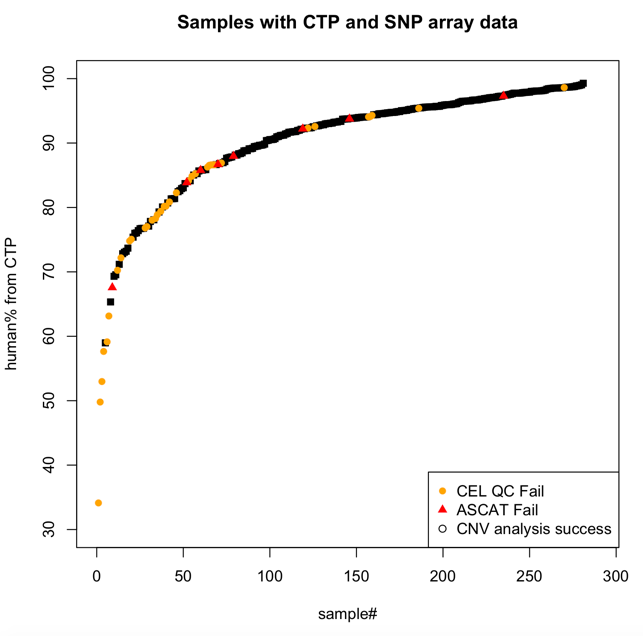


CNA

**Figure S7.**

Global CNA plots showing the overall high similarity of profiles generated by normalizing the allele-specific signal intensity of individual PDX tumors relative to 300 sex matched Affymetrix Human SNP 6.0 array samples from the HapMap project and CNA profiles generated for actual paired tumor-patient normal samples. Data for PDX model TM01594 (glioblastoma) shows results for the patient tumor (PT) and a P2 tumor. Data for PDX model TM00327 (ovarian serous adenocarcinoma) shows results for tumors from four different P0 lineages. See also Table S7.


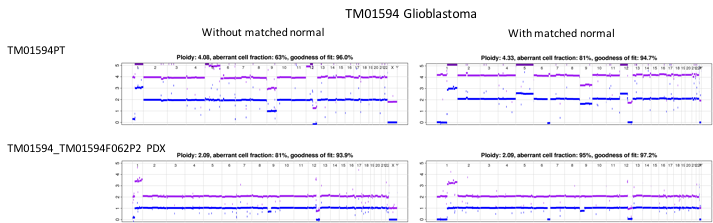


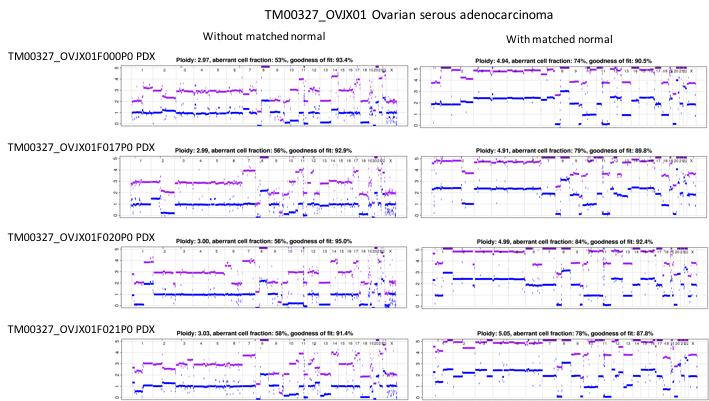


**Figure S8.**

Hierarchical clustering showing tumor type signatures of copy number gain (red) and loss (blue) for genes with GISTIC annotations in TCGA (see Table S12 and S13). [LUAD=lung adenocarcinoma; LUSC=lung squamous cell carcinoma; COADREAD=colorectal adenocarcinoma; OV =ovarian serous cystadenocarcinoma ; GBM= glioblastoma; SKCM=cutaneous melanoma; BLCA=urothelial bladder carcinoma; TNBC=triple negative breast cancer]


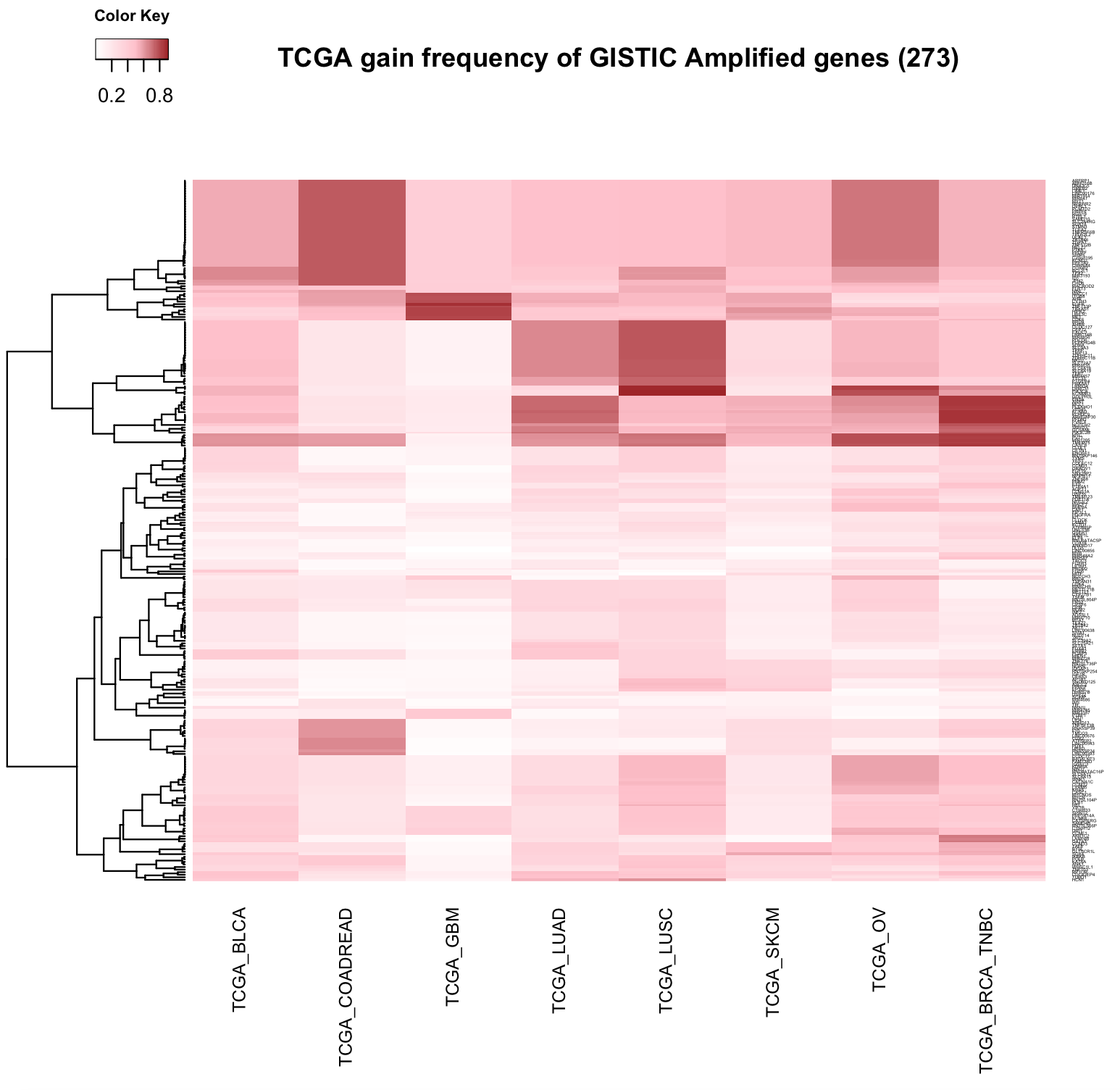

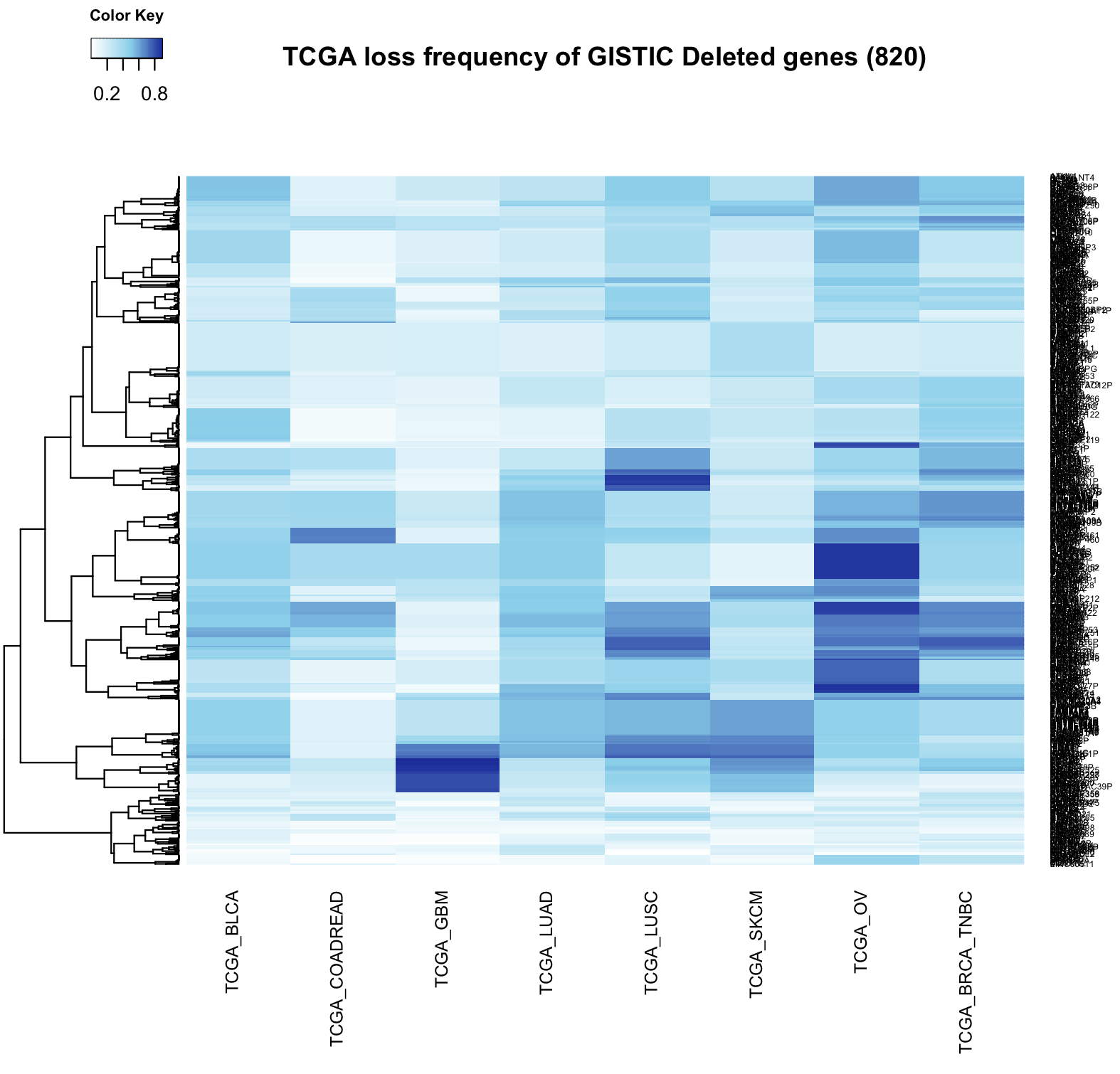


**Figure S9.**

Kernel density plots of log­_2_ copy number ratios for all amplified/deleted genes over all PDX models (N) showing the effects of different baselines for quantifying copy number (CN) aberrations. Using the diploid state as a baseline (log_2_(CN/2), plots on the right) to call CN gain and loss results in large number of genes being classified as amplified. Manually setting the boundaries for the highest peak around 0 (plots on the left) results in thresholds of low-level gain and loss as log_2_(CN/ploidy) > +0.4 and log_2_(CN/ploidy) < -0.4 respectively for PDX samples. Genes used in the analysis are those annotated in TCGA by GISTIC as being frequently amplified or deleted.


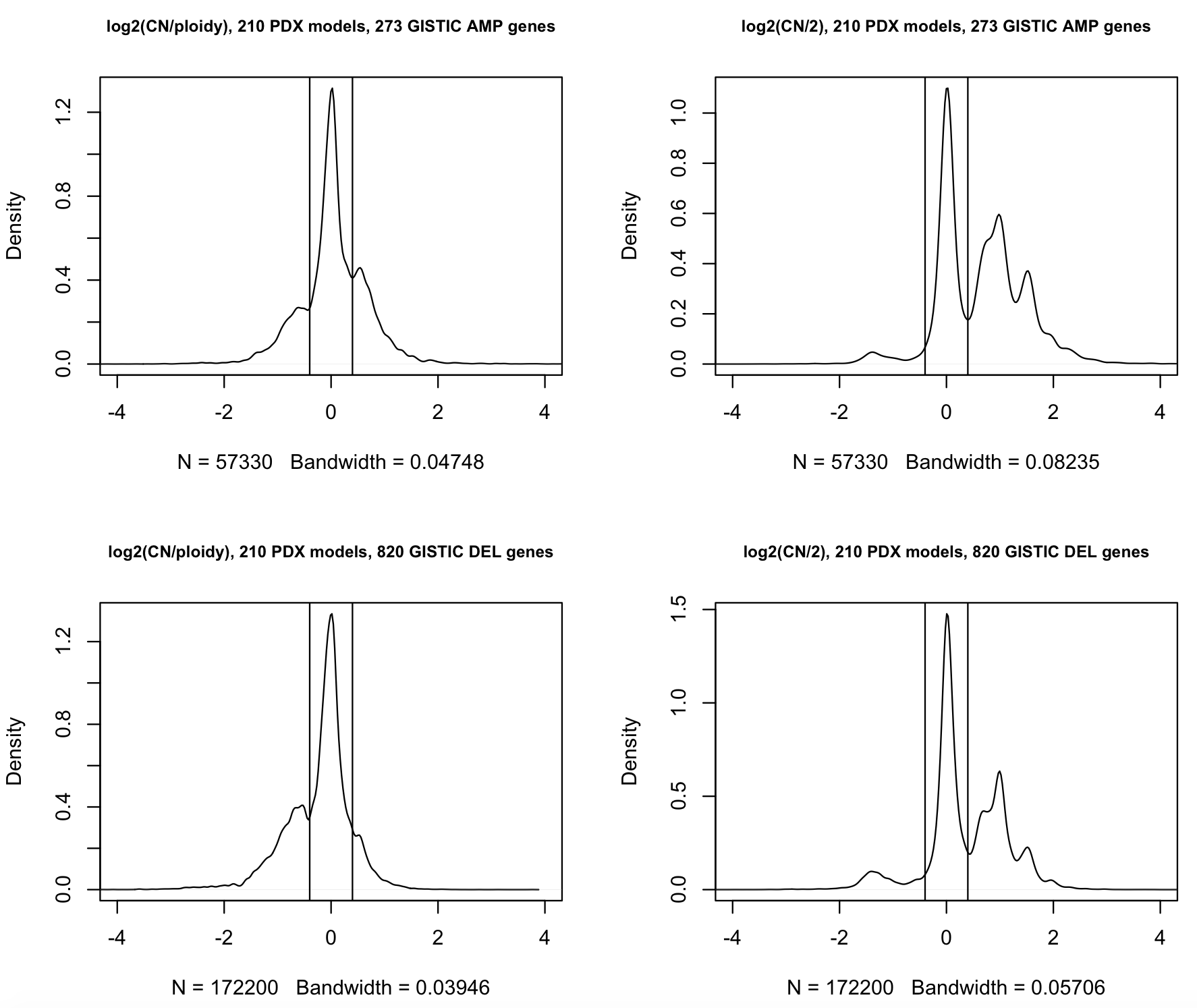


**Figure S10.**

Examples of oncogenes and tumor suppressor genes (TSG) whose expression fold changes correspond with copy number alterations in PDX tumors compared to PDX samples with normal copy number state for the same gene. The expression fold change per gene in each sample is calculated by the fold change of log2(TPM+1) relative to the mean expression across the samples with a stringent normal copy number state (-0.4 < log2(CN/ploidy) < 0.4). For comparison of expression fold change in different copy number states, a higher-level copy number gain and loss is used and defined as log2(CN/ploidy) > +1 and log2(CN/ploidy) < -1 respectively, and the normal copy number state is defined as -1 < log2(CN/ploidy) < +1. Significance in differences in expression by Student’s t-test (*=p-value < 0.005; NS=not significant). The oncogenes and tumor suppressor genes used in the analysis were based on annotations from the COSMIC Cancer Gene Census. (See Table S8)


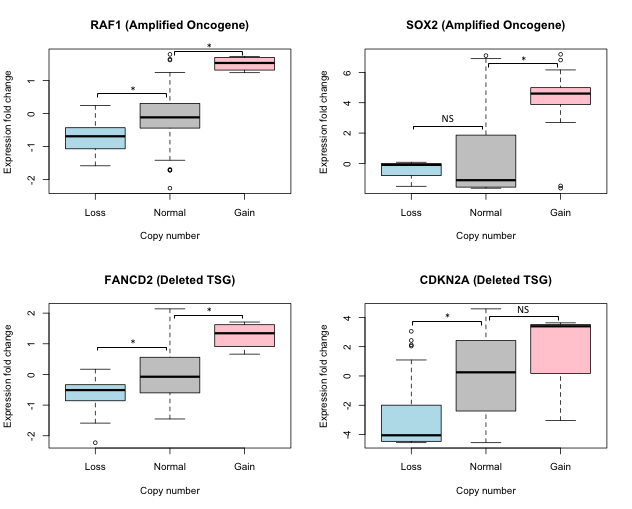


**Figure S11.**

Correlation matrices of frequencies of genes that are over-expressed (z-score of log_2_(TPM+1) > 1) or under-expressed (z-score of log_2_(TPM+1) < -1) among different tumor types in TCGA. [LUAD=lung adenocarcinoma; LUSC=lung squamous cell carcinoma; COADREAD=colorectal adenocarcinoma; SKCM=cutaneous melanoma; BLCA=urothelial bladder carcinoma; TNBC=triple negative breast cancer]

**Figure S12.**

1. Frequency of genome-wide copy number gain (red), loss (blue) and LOH (black lines) across JAX PDX models for 8 tumor types (see Table S12). The copy number gain and loss are defined as log_2_(CN/ploidy) > +0.4 and log_2_(CN/ploidy) < -0.4 respectively. LOH is defined as CN of major allele > 0.5 and CN of minor allele < 0.1.
2. Frequency of genome-wide copy number gain (red) and loss (blue) across TCGA samples for the 8 tumor types. Figures obtained from UCSC Cancer Browser, <https://genome-cancer.ucsc.edu/>. A profile specific to triple negative breast cancer (TNBC) was not available; the profile for invasive breast cancer (BRCA) is shown here.

A B


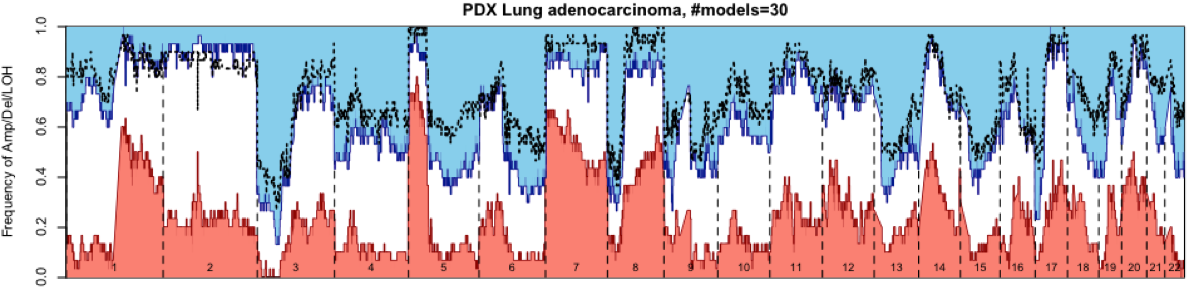

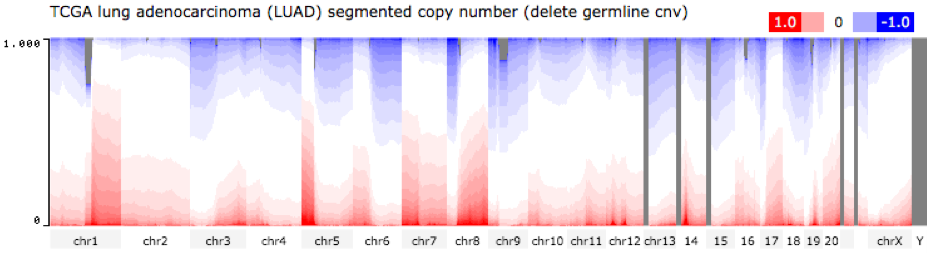


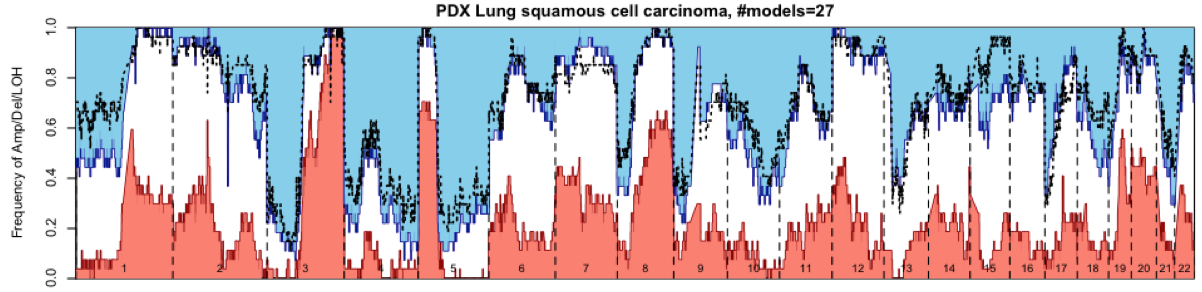

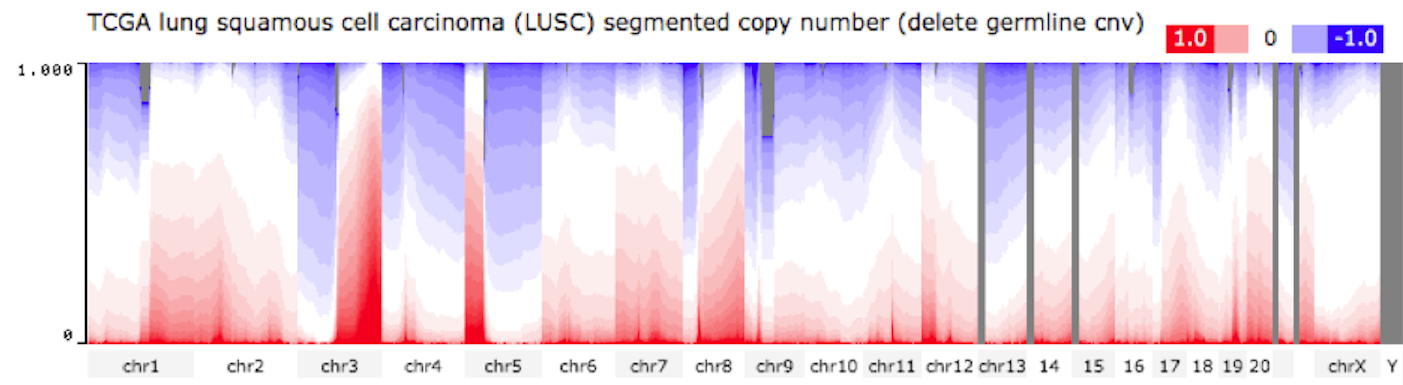


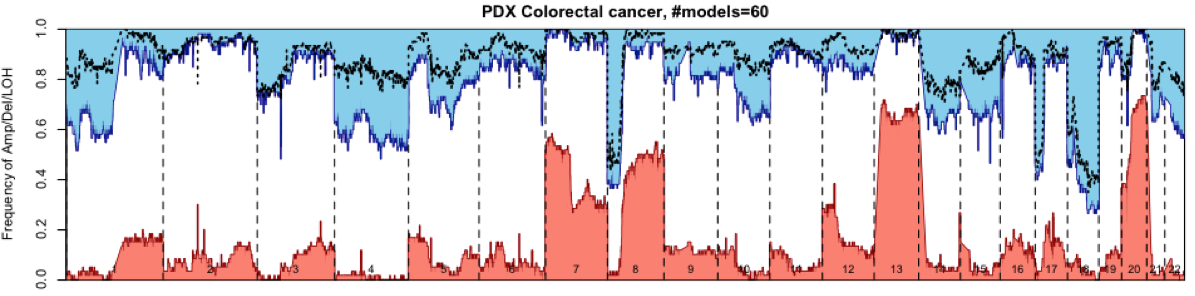

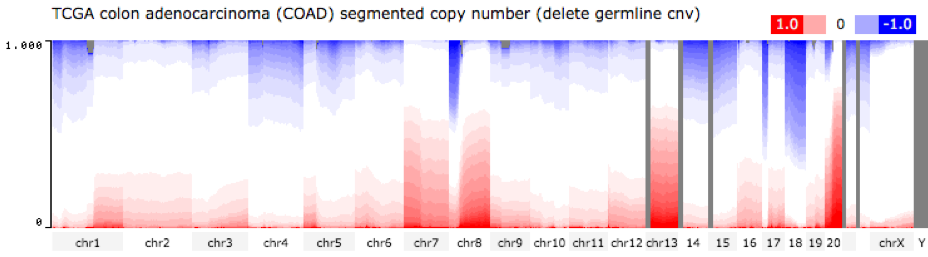


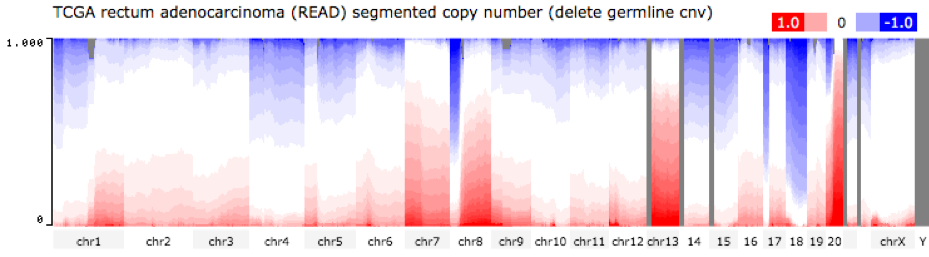


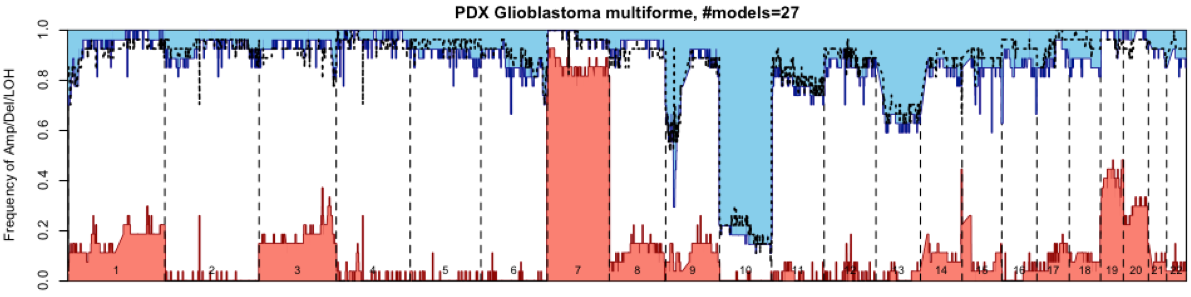

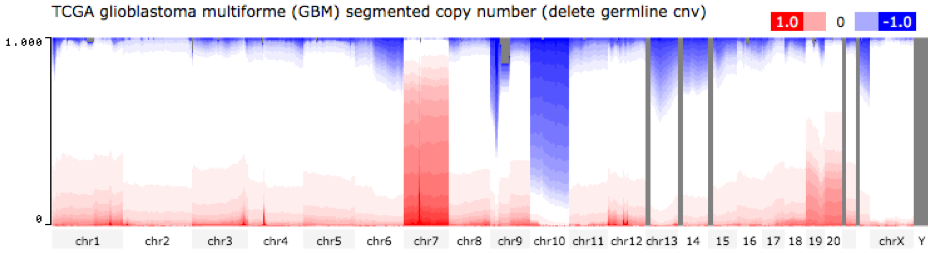


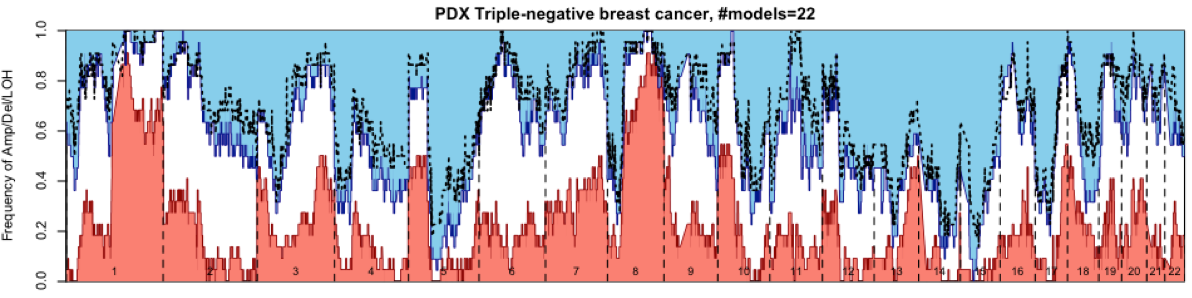

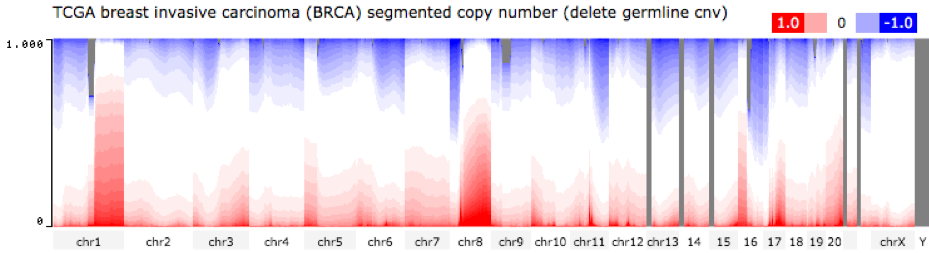


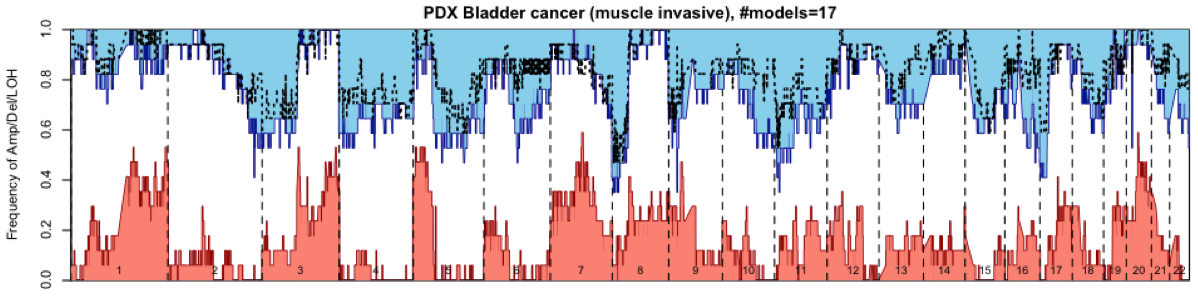

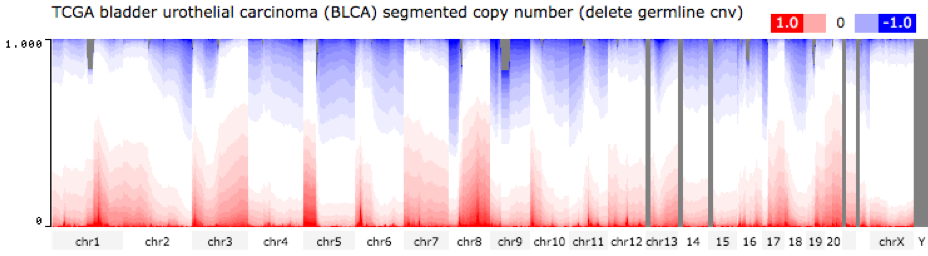


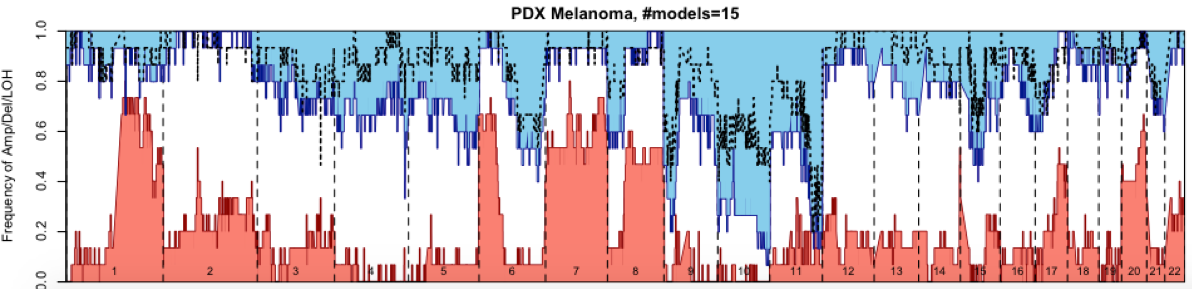

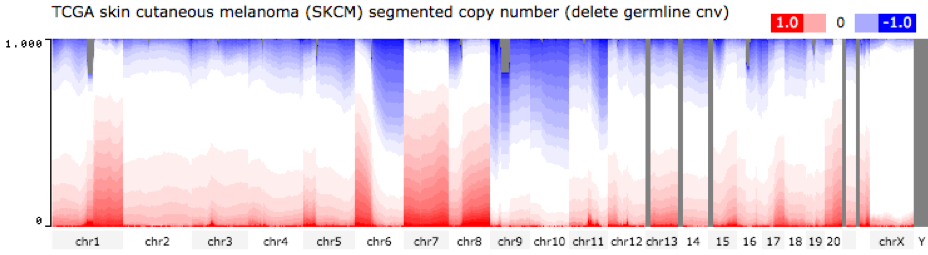


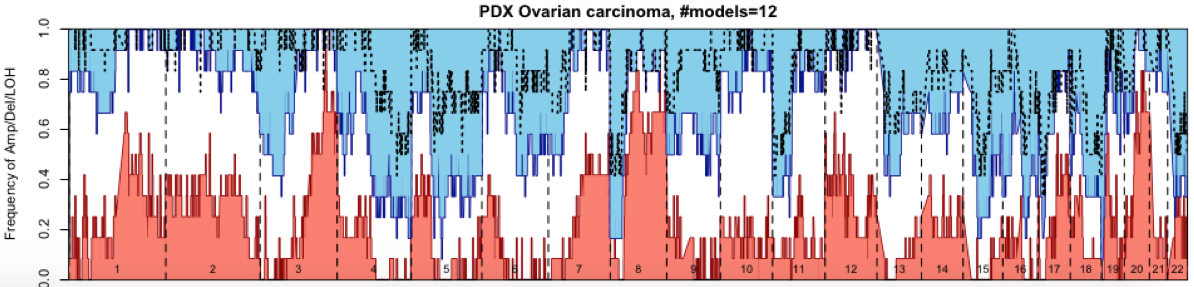

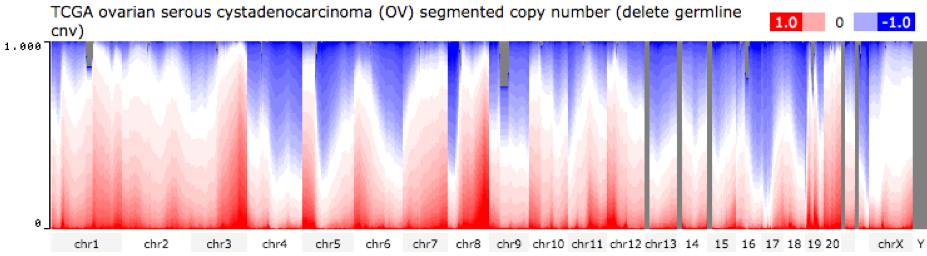


**Figure S13.**

1. Comparisons of frequencies of genes with copy number gain (red) or copy number loss (blue) between JAX PDX and TCGA tumor types when predicted ploidy is used a reference state for PDX samples. The genes included in the analysis were those predicted by GISTIC as being frequently amplified or deleted in TCGA tumor samples. The copy number gain and loss in the PDX samples was defined as log_2_(CN/ploidy) > +0.4 and log_2_(CN/ploidy) < -0.4 respectively. The copy number gain and loss in the TCGA samples was defined as > +1 and < -1 respectively. [LUAD=lung adenocarcinoma; LUSC=lung squamous cell carcinoma; COADREAD=colorectal adenocarcinoma; OV =ovarian serous cystadenocarcinoma ; GBM= glioblastoma; SKCM=cutaneous melanoma; BLCA=urothelial bladder carcinoma; TNBC=triple negative breast cancer]
2. Comparisons of frequencies of genes with copy number gain (red) or copy number loss (blue) between JAX PDX and TCGA tumors types when diploid state was used a reference state for PDX samples.

A

B

**Figure S14.**

1. Correlation matrices for genes with copy number gains (> +1, red) and copy number loss (< -1, blue) among selected TCGA tumor types [LUAD=lung adenocarcinoma; LUSC=lung squamous cell carcinoma; COADREAD=colorectal adenocarcinoma; OV =ovarian serous cystadenocarcinoma ; GBM= glioblastoma; SKCM=cutaneous melanoma; BLCA=urothelial bladder carcinoma; TNBC=triple negative breast cancer]. The genes included are those identified by GISTIC as being frequently amplified or deleted genes in TCGA tumor samples.
2. Sorted correlation coefficients based on data from Figure S15A (•) and Figure 6E (ο) showing similar trends in correlations between pairs of tumors within TCGA compared to pairs of tumors between PDX and TCGA .

A

B


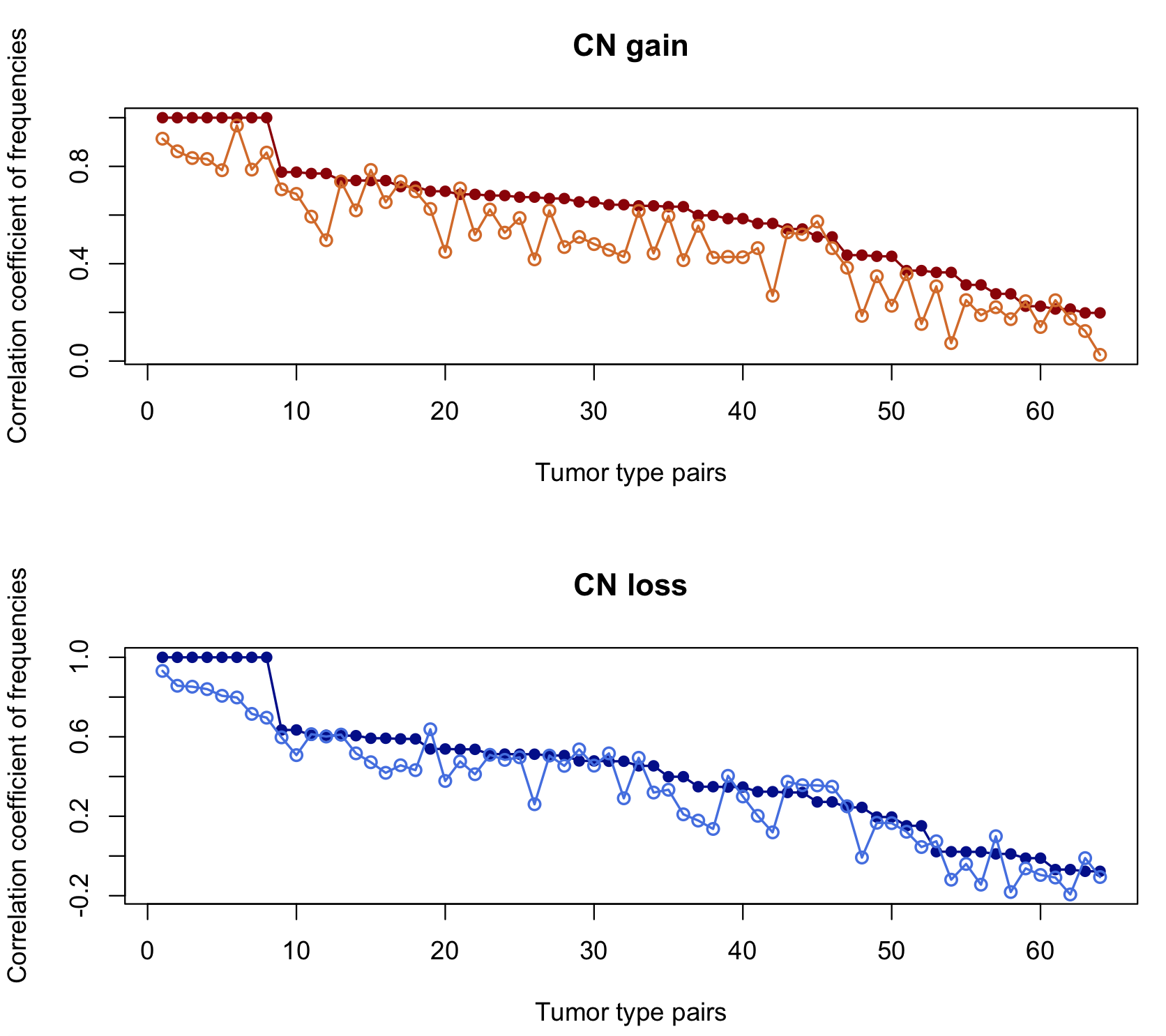


**Figure S15.**

Comparable frequencies of genomic and transcriptomic alterations in genes/pathways associated with colorectal cancer are observed between JAX PDX and TCGA colorectal tumors.

**Table S1.** *Submitted as an Excel spreadsheet in Additional File 2.*

**Table S2.** The correlation of and difference median of alternate allele frequencies between input and true positive variants for all the simulated datasets. [ALL=all variants called by the workflow; Pass= variants that meet GATK hard filter criteria as well as the minimum read depth and minimum alternate allele frequency criteria described in Methods; Output=Results from the complete variant calling workflow; NoXenome=results from variant calling workflow with the Xenome step removed; NoAltAware=results from variant calling workflow where BWA-MEM was performed without ALT-Aware].


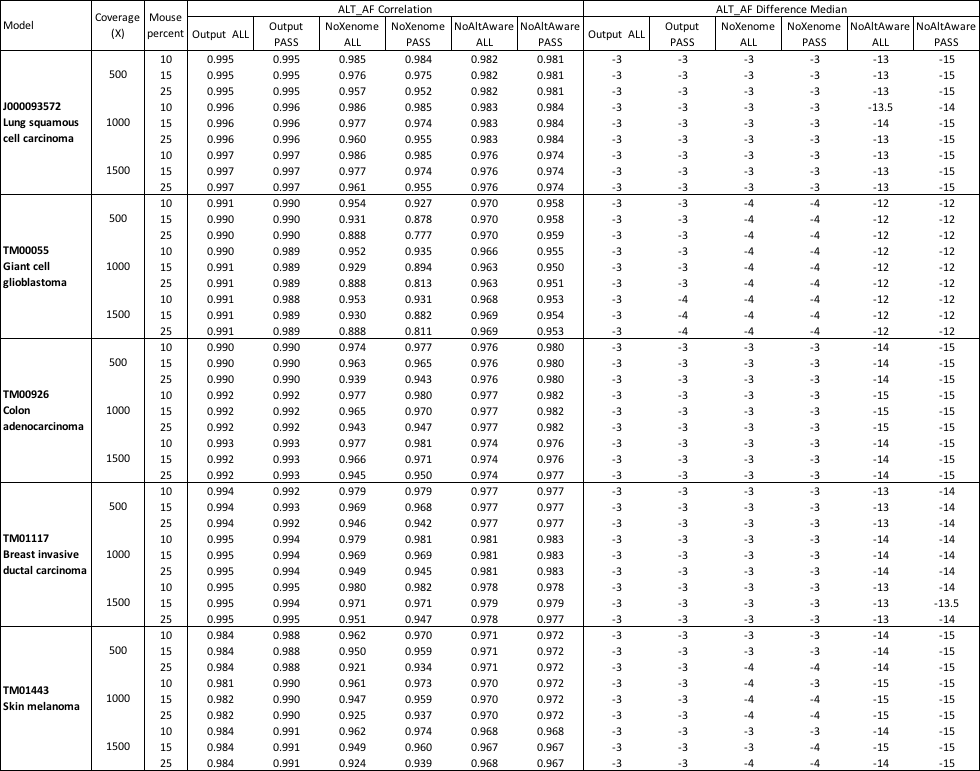


**Table S3**. Source and content of the data used to classify variants as either germline or mutation in PDX tumors that lacked paired normal control samples from the same patient.


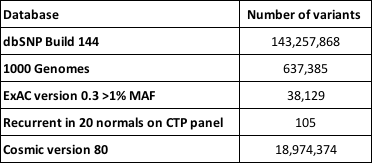


**Table S4**. The mean and median number of unique variants called by GATK for PDX CTP samples (n=383) that remain after each filtering or rescue step in the workflow. The last row shows the number of variants annotated as clinically relevant based on annotations from the JAX Clinical Knowledgebase (CKB).


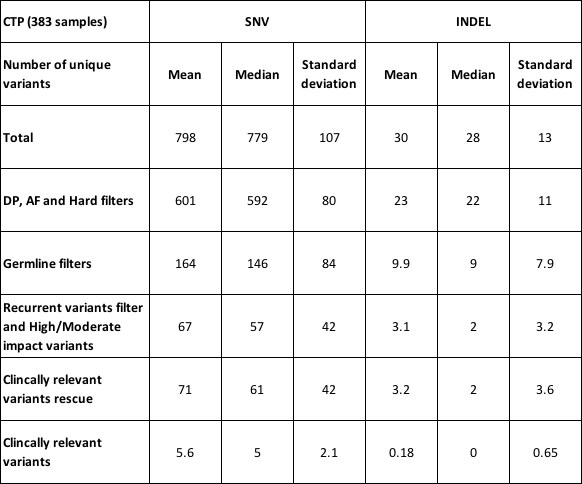


**Table S5.** Number of PDX and TCGA samples for 5 tumor types used for evaluating the performance of the variant calling workflow. The tumor types included were those that had >10 samples with CTP data in the JAX PDX resource.


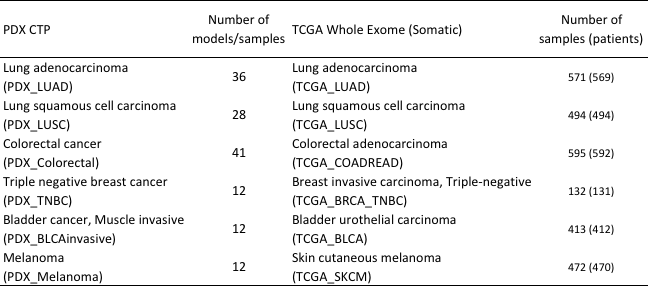


**Table S6.** Genes used to develop the expression classifier to detect EBV-associated lymphomas. Up-regulation: +1; Down-regulation: -1.


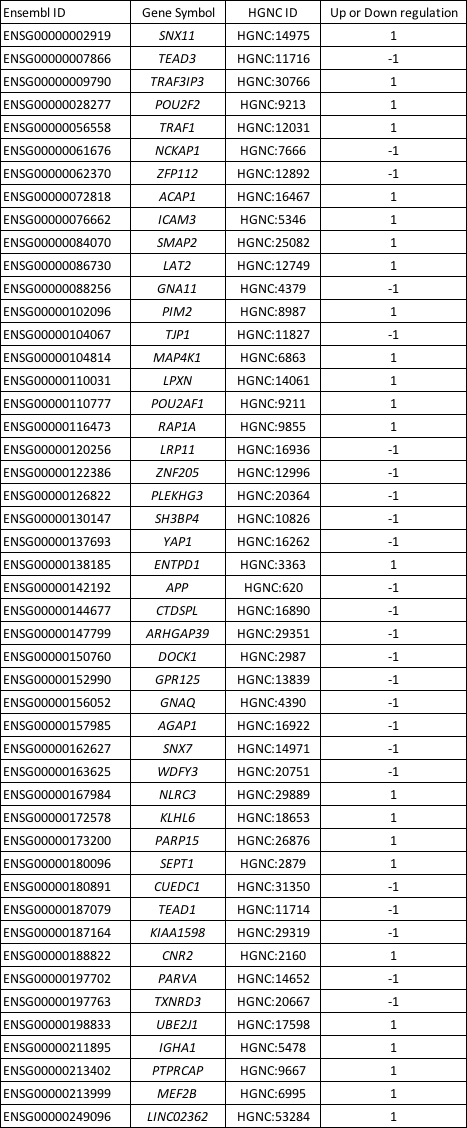


**Table S7.** Correlation coefficients **of** gene-level Copy Number Alterations (CNAs) estimates generated by normalizing individual PDX tumors with sex-matched normal sample data from the HapMap project compared to CNA values generated using available paired PDX tumor-patient normal samples.


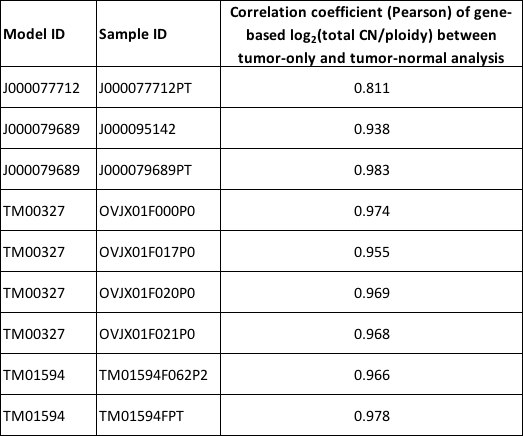


**Table S8.** Mean expression fold change of known oncogenes and tumor suppressor genes for PDX samples compared to copy number gain, loss, and normal states. The expression fold change per gene in each sample is calculated by the fold change of log2(TPM+1) relative to the mean expression across the samples with a stringent normal copy number state (-0.4 < log2(CN/ploidy) < 0.4). For comparison of expression fold change in different copy number states, a higher-level copy number gain and loss is used and defined as log2(CN/ploidy) > +1 and log2(CN/ploidy) < -1 respectively, and the normal copy number state is defined as -1 < log2(CN/ploidy) < +1. The significance of the expression fold change for each gene in the PDX samples in the gain or loss states relative to the normal state was calculated by Student’s t-test (p-value < 0.05). The oncogenes and tumor suppressor genes used in the analysis were based on annotations in the COSMIC Cancer Gene Census. (See Figure 5C and Figure S10)


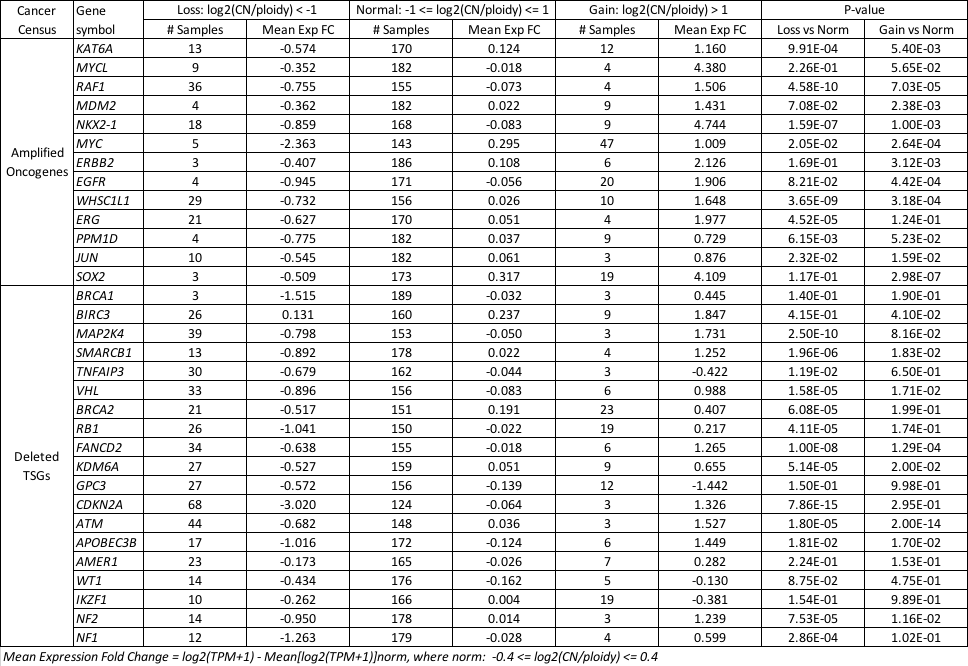


**Table S9.** Contingency table for Fisher’s Exact Test for showing counts of genes on the CTP panel with and without coding, non-silent mutations that overlap in the JAX PDX and TCGA tumor samples. For the JAX PDX samples, all CTP genes with and without coding, non-silent mutations in each tumor type cohort were counted. Because the TCGA data set has many more samples than the JAX PDX resource, only genes with coding, non-silent mutations at ≥5% and <5% frequency in each tumor type were counted. See Figure 6B for p-values computed by Fisher’s Exact Test and Table S5 for tumor type information. [COADREAD=colorectal adenocarcinoma; LUAD=lung adenocarcinoma; LUSC=lung squamous cell carcinoma; BLCA=urothelial bladder carcinoma; SKCM=cutaneous melanoma; TNBC=triple negative breast cancer]


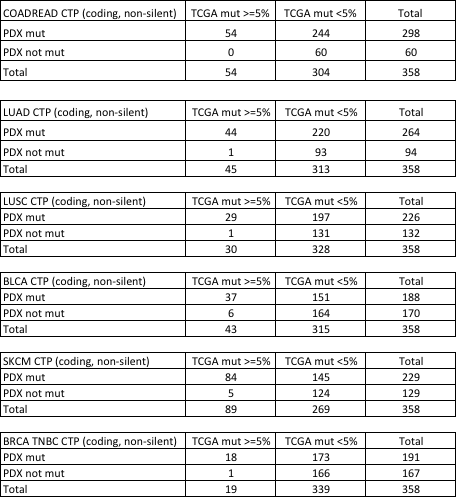


**Table S10**. Number of PDX and TCGA samples for 6 tumor types used for evaluating RNA-Seq expression profiling workflow. The tumor types included were those that had >10 samples with RNA-Seq data in the JAX PDX resource.


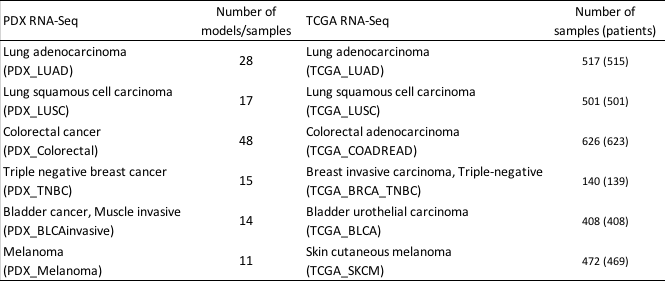


**Table S11.** Counts of up-regulated and down-regulated genes in PDX and TCGA tumors based on RNA-Seq raw count data. The numbers of genes that were up-regulated (adjusted p-value < 0.05, log (fold change) > 1) or down-regulated (adjusted p-value < 0.05, log (fold change) < -1) for each tumor type versus all other tumor types were calculated within PDX and TCGA RNA-Seq data separately. Similarity of the gene lists was calculated using the Jaccard index; significance was determined using a hypergeometric distribution.

**Table S12.** Number of PDX and TCGA samples for eight tumor types used for the analysis of copy number and loss of heterozygosity (LOH) predicted from SNP array data. The tumor types included were those that had >10 samples with SNP array data in the JAX PDX resource.


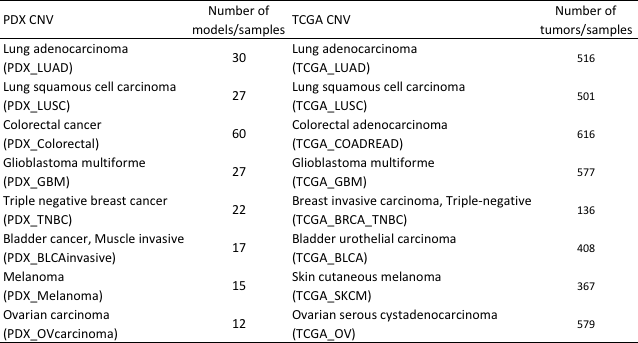


**Table S13.** *Submitted as an Excel spreadsheet in Additional File 2.*

**Table S14.** *Submitted as an Excel spreadsheet in Additional File 2.*
